# Zygotes are Persisting Organisms

**DOI:** 10.1093/jmp/jhaf039

**Published:** 2026-06-13

**Authors:** Nicholas Colgrove

**Affiliations:** Augusta University, Augusta, Georgia, USA

**Keywords:** embryos, identity, modality, miscarriage, organisms

## Abstract

Zygotes are persisting organisms. That is, zygotes are organisms and most born human beings are identical to the zygotes from which they originated. I defend these claims against recent critiques. Chunghyoung Lee, for example, argues that for any zygote, z, z may develop into one of several, numerically distinct infants. If so, then for any infant, that infant is not identical to the zygote from which he or she originated. If Lee is correct, then zygotes are like gametes, which may give rise to mature human beings, but cease to exist along the way. This, Lee claims, suggests that zygotes are not organisms. I respond that Lee (like many others) confuses zygotic parts with whole zygotes. The error lies in identifying zygotes (and embryos) with their inner contents alone. Zygotes are more than their internal parts, however, just as the reader is more than their heart, lungs, and kidneys. To advance these claims, I defend a version of “zona-essentialism,” which maintains that the zona pellucida—the membrane surrounding zygotes and early embryos—is essential to their identity during early stages of life. By distinguishing between zygotic parts and whole zygotes, I show that Lee’s (and others’) arguments fail to establish that zygotes are not organisms. I conclude by discussing practical implications for finding that zygotes are human organisms. Clinicians and researchers must embrace reality: individual human beings are routinely killed in the name of scientific advancement and reproductive autonomy. Failure to acknowledge this—e.g., by using euphemisms to obscure the truth—is problematic.

## I. INTRODUCTION

Following Chunghyoung Lee, let “zygote” refer to “a 1-cell embryo that is formed as a result of the fusion of a human ovum and sperm” (2022, 297). All zygotes are embryos. Not all embryos are zygotes, however, since many embryos possess more than one cell. Philosophers often argue that human zygotes are not organisms. For example, van Inwagen (1990) claims that human organisms begin to exist between 4 and 24 days postconception. Lee claims that human organisms begin to exist around “day 8 after fertilization” at the earliest (2022, 322). Baker (2007) asserts that human organisms begin to exist only after the possibility of twinning is eliminated. This, Condic (2020) notes, occurs around 14 days postconception. Smith and Brogaard (2003) argue that human organisms begin to exist around 16 days postconception at the latest. Brown (2019) goes further, arguing that human organisms do not begin to exist until 9 weeks postconception at the earliest. Kingma (2020) goes furthest, claiming that human organisms do not begin to exist until they have completed birth (i.e., around 20-22 weeks postconception at the earliest). On any such view, zygotes are not organisms.^1^

By contrast, neurobiologist Maureen Condic argues that our best “scientific evidence supports the conclusion that a zygote is a human organism” (2008, ix). Philosophers may recoil at Condic’s claim since *metaphysical* claims are in dispute, which cannot be settled by scientific inquiry. Nonetheless, in this essay, I defend Condic’s view: zygotes are organisms. Moreover, I argue that most born human beings are identical to the zygotes from which they originated.^2^ Let this two-part thesis—that (1) zygotes are organisms and (2) most born human beings are identical to the zygotes from which they originated—be the “zygotes-are-persisting-organisms” thesis (or ZAPO). By calling a zygote a “persisting organism,” I do not mean that the organism persists *as a zygote* across time. Rather, the organism persists from its stage as a zygote, to a fetus, newborn, etc., over time.

ZAPO has been defended by others. Miller and Pruss argue that “the best reading of the metaphysics of fertilization is that the oocyte and spermatid come together to produce a new organism, the zygote” and “the zygote is identical to the mature human organism that it grows into” (2017, 542). ^3^ My defense differs from others, however, as I argue for ZAPO along slightly different lines and against more recent objections. Specifically, Lee’s (2022) rejection of ZAPO has gone unchallenged to date, despite being among the most rigorous criticisms of ZAPO.^4^ Thus, I focus heavily on responding to Lee. My response also exposes errors that pervade other works on the metaphysics of embryos: philosophers routinely confuse embryonic *parts* with whole embryos. Correcting this error shows why ZAPO is defensible. ZAPO’s defensibility, in turn, has important practical implications for clinical practice and research.

In Sections II and III, I outline Lee’s argument against ZAPO. Section II identifies the stakes of the debate. Section III examines the details of Lee’s argument. In Section IV, I explain why Lee’s argument fails. In short, Lee confuses part of an embryo (its inner cell mass) with the embryo itself. That is, if we grant that embryos are more than their inner cell mass, then Lee’s argument against ZAPO fails. In Section V, I respond to two objections that Lee might raise against my critique. Next, in Section VI, I defend the claim that embryos are more than their inner cell mass. Specifically, I defend a version of “zona-essentialism,” the view that a de facto zona pellucida—where the zona pellucida is the membrane that separates zygotes and early embryos from the outside world—is an essential part of early embryos.^5^ Zona-essentialism draws criticisms from biologists and metaphysicians. So, in Section VII, I defend it against objections. Doing so completes my defense of ZAPO. In Section VIII, I use this defense to explain why other critiques of ZAPO fail.

Finally, in Section IX, I discuss practical implications for clinical practice and research. If ZAPO is correct, then changes are warranted with respect to clinical guidelines (e.g., concerning abortion policy and management of miscarriage). Changes are also warranted with respect to clinical trial guidelines (e.g., concerning the use of embryos in research). Essentially, if ZAPO is proven correct, then medical and scientific communities must contend, openly and honestly, with the fact that human organisms are routinely killed in the name of “reproductive autonomy” and “scientific innovation.” An honest discussion, moreover, would require admitting that current practices (that involve destruction of embryos) cannot be justified on medical or scientific grounds alone. Indeed, any defense of these practices must invoke contentious philosophical claims. Clinicians and scientists possess no special expertise or authority—qua clinicians and scientists—when it comes to philosophical disputes. So, when determining which practices are justifiable, medical and scientific experts must defer to other experts. I expect clinicians and researchers to resist these suggestions. They may think my claim is an attempt to usurp their authority (e.g., to determine which practices are justifiable). In this context, however, the medical and scientific communities have often overstepped their bounds. Thus, what may look like a “land grab” by philosophers is simply the returning of stolen territory to its rightful owners.

## II. LEE’S ARGUMENT AGAINST ZAPO: UNDERSTANDING THE STAKES OF THE DEBATE

Beginning with a discussion of ZAPO, Lee (2022) argues that human zygotes are not organisms. His argument relies on the claim that no adult is identical to the zygote from which he or she originated. So, by refuting ZAPO’s second conjunct, Lee challenges its first. In this section, I sketch Lee’s main argument and its implications. Next (in Section III), I carefully unpack one of Lee’s key premises. This enables us to see (in Section IV) why Lee’s argument fails.

First, Lee defends “Developmental Plasticity” which states that “a human zygote that naturally develops into an infant without twinning could have naturally developed into a numerically different infant without twinning” (2022, 322). According to Lee (2022), every zygote is developmentally plastic. For now, suppose that is correct. It follows that for any zygote, *z*, *z* could naturally develop into one infant, *A*, or a numerically distinct infant, *B*. This means that *z* could become *A*, *z* could become *B*, and *A* ≠ *B*. Since *A* ≠ *B*, it follows that neither *A* nor *B* is identical to *z*. After all, *A* has no more claim to be *z* than does *B*. Thus, it cannot be that just *one* of the infants is identical to *z*. Either both *A* and *B* are identical to *z* or neither is.

Both infants cannot be identical to *z*. To demonstrate, assume (for reductio) that both *A* and *B* are identical to *z*. Identity is transitive. So, if *z *=* A* and *z *=* B*, then *A *=* B*. Given our assumption—that every zygote is developmentally plastic—we already know that *A* ≠ *B*. If Lee is correct about developmental plasticity, therefore, then on pain of contradiction, both *A* and *B* cannot be identical to *z*. This leaves only one possibility: neither *A* nor *B* is identical to *z*. If so, then it follows that “every zygote is numerically distinct from any actual or possible infant, child, or adult (or any full-fledged human being)” (Lee, 2022, 318). Put simply, for any zygote, *z*, and for any infant *I* that *z* develops into, *z* ≠ *I*. This conclusion has three major implications.

First, and straightforwardly, it means that no infant (and no adult) is identical to the zygote from which they originated. Human beings reading this essay are not identical to the zygotes from which they originated.^6^ Rather, in the past, some zygotes ceased to exist and the humans reading this essay began to exist. Whenever readers began to exist, Lee (2022) argues, it cannot be before eight days postconception.

Second, if no infant (and no adult) is identical to the zygote from which they originated, then this “strongly suggests that a zygote is not a human being” (Lee, 2022, 322).^7^ This is surprising. Now as Ford argues, a human being is “a living individual with a human nature” that possesses “the inherent active potential to develop toward human adulthood without ceasing to be the same ontological individual” (1988, 67, 84-85). If so, then human beings naturally develop across time from infants and children into adults. Condic echoes this point, asserting that human beings undergo an “orderly sequence of maturation that results in a characteristic adult form” (2020, 9). If zygotes are never identical with human infants, children, or adults, however, then zygotes *cannot* participate in most of “the human life-cycle.” Nor can zygotes develop into “a characteristic adult form.” Instead, zygotes—and, likely, the multicellular embryos that originate from zygotes—cease to exist at some point before giving rise to numerically distinct entities. This makes zygotes akin to “an ovum and sperm, which go out of existence” well before infancy and adulthood (Lee, 2022, 319). Thus, Lee’s argument undermines any claim that killing zygotes is morally wrong when the wrongness of killing zygotes is grounded in their purported status as “human beings.” Calum Miller, for example, argues that killing embryos is objectionable because it violates the “respect” owed to human beings (2023, 235). If zygotes are not human beings, however, then Miller’s argument does not apply.

Third, even if zygotes *are* human beings, if a zygote is never identical with any infant (or adult), then killing zygotes still seems relatively unobjectionable. To illustrate, Marquis (1989) famously argues that killing zygotes is wrong because it deprives them of a “future-like-ours”: future experiences, relationships, and happiness, which are all tremendously valuable. If zygotes are never identical with the infants or adults they develop into, however, then zygotes cannot have a future-like-ours. Whether they are killed or not, zygotes always cease to exist long before attaining goods associated with a future-like-ours. So, if killing a human being is wrong because it deprives them of a future-like-ours, then killing zygotes seems unobjectionable, since they have no future-like-ours to lose.^8^ Hence, Lee’s argument against ZAPO has significant moral implications.

In response to Lee, one could dispute what it means to be a human being (as articulated by Ford and Condic). Or one could follow Hershenov (2023) in arguing that killing zygotes is impermissible, even if it is metaphysically impossible for them to develop into infants and adults.^9^ I prefer a more focused strategy. Each of the above implications stem from Lee’s defense of Developmental Plasticity: the claim that “a human zygote that naturally develops into an infant without twinning could have naturally developed into a numerically different infant without twinning.” Thus far, I granted this claim and described what would follow. Yet, if Lee is mistaken about Developmental Plasticity, then his entire argument collapses. My task, therefore, is to show why Lee is mistaken about Developmental Plasticity.

## III. LEE’S DEFENSE OF DEVELOPMENTAL PLASTICITY

Lee’s defense of Developmental Plasticity is complicated. The crux of his argument is the claim that for any zygote, *z*, and for any infant *I* that originates from *z*, *z* ≠ *I*. This claim rests on several observations about embryology and modality.

First, let us examine embryology. Successful conception results in “the fusion of an ovum and a sperm” which produces “a zygote” or “1-cell-embryo.” Next, the zygote undergoes a series of “cleavages,” becoming a 2-celled embryo, 4-celled embryo, and so on. Each of these cells is referred to as “blastomere.” While the embryo develops, “a process called compaction occurs: the blastomeres huddle together … and one or two of them are pushed to the inside.” Soon after, “two types of cells become clearly distinguished: the inner cell mass coming from the inner blastomeres … and the trophoblast … which is the outer layer coming from the outer blastomeres” (Lee, 2022, 303). Lee writes, “when an infant is born, her cells come from the inner cell mass, while the trophoblast produces no tissues of the infant but only part of the placenta” (2022, 303). To quantify this, at the 16-cell stage, approximately 6 blastomeres form the inner cell mass, while the other 10 blastomeres serve as outer cells. Curiously, at the 16-cell stage, inner and outer blastomeres can be rearranged. Inner blastomeres can be moved to the outside and vice versa. This is where modality becomes relevant.

Regarding modality, Lee imagines five possible worlds. In the first world (the actual world), the following occurs:

*Actual World*: “A zygote *z* is formed … and naturally develops into a singleton infant, Lea.” At the 16-cell stage, blastomeres *B*_1—_*B*_6_ form *z*’s “inner cell mass,” whereas the remaining blastomeres, *B*_7—_*B*_16_ “yield the trophoblast.” (Lee, 2022, 305)

In the *Actual World*, an unremarkable zygote develops into an infant. Both the zygote and the singleton into which it develops never undergo twinning or anything unusual. Things could have been different, however. To illustrate, Lee introduces four other possible worlds (*W_1—_W_4_*, respectively). In the first alternative world (*W_1_*) the following events occur:

*W_1_*: *z* develops into a 16-cell embryo. *B*_1—_*B*_6_ form *z*’s inner cell mass; the remaining blastomeres (*B*_7—_*B*_16_) move to the outside. Next, clinicians remove eight blastomeres from *z*—namely, *B*_9—_*B*_16_ and destroy them. Clinicians then produce eight copies of *B_8_* and use these copies to replace *B*_9—_*B*_16_. From this point, the 16-cell embryo continues to grow, uninterrupted. Eventually, this process results in an infant named “Thea” being born. (Lee, 2022, 306)

Compare the *Actual World* to *W_1_*. Regarding Lea and Thea, Lee (2022) argues, each infant’s cells come entirely (or, at least, almost entirely) from blastomeres *B*_1—_*B*_6_. So, it seems plausible that Lea and Thea are identical: “Lea” and “Thea” refer to the same individual across two different possible worlds. After all, Lee states, when comparing the events of the *Actual World* to *W*_1_ “the series of the stages including zygote *z*, [*B*_1—_*B*_6_], the main body … of the fetus, and the body of the infant at birth in *W*_1_ is completely identical (molecule by molecule, cell by cell, organ by organ, and so on)” (2022, 309). Hence, Lea = Thea.^10^

Things change in the next possible world (*W_2_*), however.

*W_2_*: *z* develops into a 16-cell embryo. *B*_1—_*B*_6_ form *z*’s inner cell mass; the remaining blastomeres (*B*_7—_*B*_16_) move to the outside. Next, clinicians remove eight blastomeres—specifically, *B*_9—_*B*_16_ and keep them. Clinicians then produce eight copies of *B_8_* and use these copies to replace *B*_9—_*B*_16_. From this point, the 16-cell embryo continues to grow, uninterrupted. Eventually, this process results in an infant named “Thea” being born (exactly as occurred in *W_1_*). Meanwhile, since *B*_9—_*B*_16_ have been preserved, clinicians join those blastomeres with eight more copies of *B_8_*. *B*_9—_*B*_14_ form the inner cell mass of the resulting 16-cell embryo. From there, the embryo develops uninterrupted. The process results in an infant, “Shae,” being born. (Lee, 2022, 307)

Infants Thea and Shae are numerically distinct. After all, they are “ontologically independent”, in that one could cease to exist without affecting the existence of the other. Second, “their bodies are composed of completely different matter, cells, and organs” (Lee, 2022, 307). So, Thea ≠ Shae. Since *W_1_* taught us that Lea = Thea, it follows that Lea ≠ Shae.

Lee’s defense of Developmental Plasticity requires imagining one more possible world: W_*4*_.^11^

*W_4_*: *z* is formed and reaches the 16-cell stage. Natural forces push *B*_9—_*B*_14_ to the center, and so, *B*_9—_*B*_14_ form the inner cell mass. Next, *z* develops uninterrupted. This process results in an infant, “Mae,” being born. (Lee, 2022, 307-308).

*W_4_* describes a real possibility. As Lee observes, “the fate of blastomeres is not determined solely by the intrinsic properties of the embryo at its 1-cell stage” (2022, 308). When comparing the *Actual World* to *W_4_*, however, Lea (from *Actual World*) and Mae (from *W_4_*) have bodies “composed of different matter, cells, and organs coming from different inner cell masses.” As such, it seems like Lea and Mae are not identical.^12^ Furthermore, Mae arose from *B*_9—_*B*_14_, just like Shae (in *W_2_*). Thus, it seems that Mae = Shae.

Recall, we learned from *W_2_* that Thea ≠ Shae. We also know from *W_1_*, that Lea = Thea. It follows that Lea ≠ Shae and, more importantly, Lea ≠ Mae. The zygote, *z*, therefore, may naturally develop into Lea or Mae. Since Lea and Mae are numerically distinct (non-identical) individuals, however, it follows that *z* is Developmentally Plastic. That is, *z* could naturally develop into one of several numerically distinct infants, *even without twinning*. Since *z* may develop into one of several numerically distinct infants, it follows that any resulting infant cannot be identical with *z*. Either all resulting infants are identical to *z*—which, we have seen, results in a contradiction—or none of them are. Lessons about Lea, Thea, Shae, and Mae generalize. Thus, Lee’s case for Developmental Plasticity rests: for any zygote, *z*, and for any infant *I* that originates from *z*, *z* ≠ *I*.

## IV. WHY THE ARGUMENT FOR DEVELOPMENTAL PLASTICITY FAILS

Having looked carefully at Lee’s argument, we can expose its flaws. Like Lee, I discuss embryology before attending to modality.

### On Embryology

**Fig. 1.**
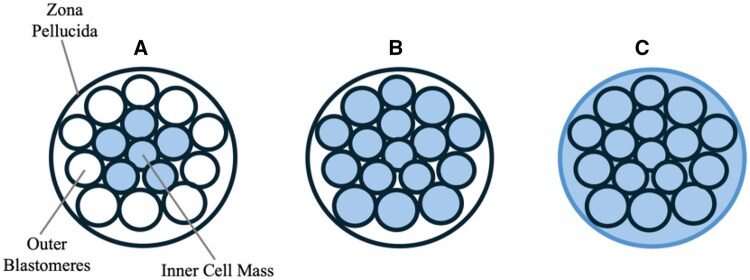
Three ways to identify relevant embryonic parts.

First, regarding embryology, there are at least three things to consider when discussing embryos at the 16-cell stage: their inner cell mass, outer blastomeres, and zona pellucida (henceforth, “zona”). The inner cell mass is composed of blastomeres that have been pushed to the center of the embryo. Outer blastomeres surround the inner cell mass and eventually give rise to the trophoblast (a precursor to the placenta). The zona, Sadler writes, “is a glycoprotein shell surrounding the egg that facilitates and maintains sperm binding” (2019, 41). That is, the zona surrounds both an unfertilized ovum and serves as “an outer membrane to the cells of the embryo” during early development.^13^ Regarding 16-cell embryos, we should ask, which parts are relevant to their identity across time?^14^ There are at least three ways to answer this question (see fig. 1).

Figure 1 illustrates three ways of thinking about 16-cell embryos, where blue shading highlights parts of embryos that may be regarded as relevant to their identity over time.

On Option (A), an embryo’s inner cell mass is judged to be especially relevant to its identity. This may seem intuitive. As Lee notes, the outer blastomeres form the trophoblast (and then placenta) which “plays only a supportive, not constitutive role in the formation of the infant’s body” (2022, 311). So, if I were to look back at the 16-cell embryo that “grew into” me, I might identify “me” with the inner cell mass alone, while the other parts formed only temporary, “supportive” apparatuses.

Option (A) also looks appealing, given lessons from the *Actual World* and *W_1_*. In *W_1_*, all of *z*’s outer blastomeres were exchanged, but this did not seem to alter the identity of the singleton (Thea) produced by *z*. That is, Lea—who resulted from the unaltered embryo in the *Actual World*—plausibly remained identical to Thea in *W_1_*, even though the embryo that became Thea had all its outer blastomeres exchanged. Relatedly, it seemed that when blastomeres *B*_9—_*B*_14_ formed the inner cell mass of the embryo in *W_4_*, the resulting infant, Mae, was numerically *distinct* from Lea. If so, then yet again, the inner cell mass seems especially relevant to an embryo’s identity over time.

On Option (B), one could argue that *all* blastomeres (both inner and outer) are relevant to an entity’s identity. This is not to say that each blastomere is required to preserve identity. Maybe a mere majority is needed. Either way, Option (B) suggests that more than the embryo’s inner cell mass is relevant to its identity. Accepting (B) would cast doubts on Lee’s claims about Lea and Thea. In the *Actual World*, *z* develops into an embryo with blastomeres *B_1_—B_16_*. If, at the 16-cell stage, an embryo’s identity depends on all sixteen blastomeres (or, perhaps, a simple majority of them), then it seems that, contra Lee, the embryo’s identity will *not* survive the removal of blastomeres *B_7_—B_16_*. If so, then contra Lee, Lea ≠ Thea.

On Option (C), at the 16-cell stage, an embryo’s blastomeres (both inner and outer) and zona are all relevant to its identity. There are excellent reasons to include the zona as critical to the identity of 16-cell embryos, as I argue in Section VI. For now, let us simply assume the following: a 16-cell embryo’s identity cannot survive the destruction of its de facto zona—that is, it cannot survive the loss of the zona or whatever is performing the *function* of the zona—at that stage of development.^15^ Call this assumption, “zona-essentialism.” On zona-essentialism, the identity of a 16-cell embryo depends, in part, on its de facto zona. So, even if *parts* of a 16-cell embryo—namely, its inner and outer blastomeres—can survive destruction of the de facto zona, the embryo’s identity cannot. If zona-essentialism is right, then Lee’s argument for Developmental Plasticity fails. Here is why.

### On Modality

When discussing modality, Lee defends two sets of identity statements:

 Identity Statements, Set 1:

  Lea = Thea

  Thea ≠ Shae

   So, Lea ≠ Shae

 Identity Statements, Set 2:

  Mae = Shae

   So, Mae ≠ Thea

   And, Mae ≠ Lea

Plausibly, on zona-essentialism, Lea = Thea. In *W_1_*, only the embryo’s outer blastomeres were replaced; the inner blastomeres and zona remained intact. Thea, therefore, seems like Lea as if Lea had received a blood transfusion and donor organs. As with organ donation, some parts of an entity’s body are replaced, but the individual’s identity survives those changes. This reveals why we must distinguish zona-essentialism from the claim that a 16-cell embryo’s identity is changed whenever there is alteration to any of its blastomeres. That is, zona-essentialism is compatible with the claim that embryos may survive some changes to their blastomeres.

Next, it seems correct that Thea ≠ Shae. Shae, recall, is composed (in part) of *B*_9—_*B*_16_ (plus eight copies of *B_8_*). Shae does not begin to exist in a vacuum, however. To function at this stage of development, the extracted and copied blastomeres must be placed within a new, distinct zona. Something like this occurs with the creation of a “three-parent embryo,” where the inner content of one embryo is placed within the distinct, hollowed-out zona of another embryo.^16^ On zona-essentialism, the transfer of blastomeres *B*_9—_*B*_16_ into a new zona suggests that the new entity is not identical to *z*. So, given zona-essentialism, we should conclude that Shae ≠ Thea. Thus, zona-essentialism plausibly affirms the first set of Lee’s identity claims, albeit for different reasons than he gives.

On zona-essentialism, the second set of identity claims are contentious. It seems *false* that Mae = Shae. The embryo that became Mae, recall, came to exist in *W*_4_ when *z* was left alone and blastomeres *B*_9—_*B*_14_ naturally formed the embryo’s inner cell mass. By contrast, the embryo that became Shae in *W*_2_ began to exist when blastomeres *B*_9—_*B*_14_ were *extracted* from *z* and placed into a new zona. On zona-essentialism, doing this may be akin to removing the heart, lungs, kidneys, etc., of one human being and placing them into the otherwise “hollowed-out” body of another. The individual parts from the original individual remain intact. We might even say that those parts *survive* the process. But those parts are disintegrated from the original individual and then integrated into a new individual. So, in *W*_2_, when blastomeres *B*_9—_*B*_14_ are extracted from one entity and placed into a new zona, the resulting entity is not identical to the original. This means that Mae ≠ Shae, contrary to Lee’s claims.

More importantly, on zona-essentialism, plausibly Lea = Mae. In the *Actual World*, which gives rise to Lea, blastomeres *B*_1—_*B*_6_ form the embryo’s inner cell mass. In *W_4_*, which gives rise to Mae, *B*_9—_*B*_14_ form the embryo’s inner cell mass. Granting that Lea and Mae are (at least) phenotypically quite different, on zona-essentialism, they plausibly remain numerically identical. This implication is not as strange as Lee suggests. Suppose that blastomeres can rearrange themselves. It follows that a single zygote, *z*, may *express itself* in radically different ways across possible worlds. In the *Actual World*, *z* expresses itself as Lea, whereas in *W_4_*, *z* expresses itself as Mae. But despite differences in expression—and resulting differences in phenotype—Lea and Mae grow continuously from the same *complete* embryo. So, it seems Lea = Mae.

**Fig. 2.**
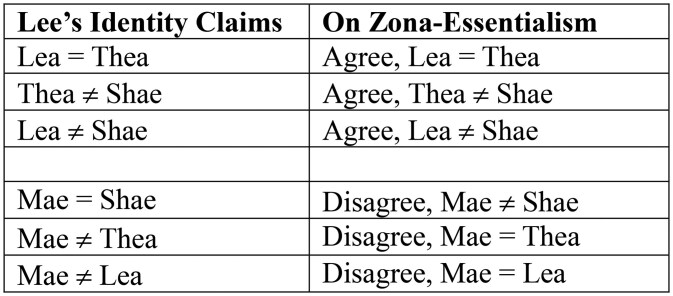
Competing claims about identity.

Here is a summary of differences between Lee’s view and those plausibly implied by zona-essentialism (see fig. 2).

If zona-essentialism is correct, then Lee’s argument for Developmental Plasticity fails. Recall Lee’s argument: For a zygote, *z*, *z* may develop into Lea or develop into Mae. Lee reasons that since Lea and Mae are *numerically distinct* (non-identical) individuals; however, it follows that *z* is Developmentally Plastic. This argument generalizes to all zygotes. Now if every zygote is developmentally plastic, then ZAPO is false.

Zona-essentialism, by contrast, plausibly implies that, contra Lee, Lea and Mae are numerically identical. The crux of his argument—that Lea and Mae are numerically distinct individuals—is false. Lea and Mae are the same being across different possible worlds (albeit radically different expressions of the same individual). If so, then Lee’s main argument for Developmental Plasticity is unsound and his main argument against ZAPO fails.

## V. OBJECTIONS: IDENTITY AND UNINTERRUPTED DEVELOPMENT

Lee anticipates a response like mine and recognizes that someone could deny:

… both that Thea is Lea in *W_1_* and that Mae is not Lea in *W_4_* by appealing to the following intuition. Let us say that an embryo *continuously* develops into an infant *without disruption* if it develops into exactly one infant without any fission, fusion, rearrangement, partial loss or destruction, and so on. (2022, 312)

Call this the “Uninterrupted Development” objection. Lee’s imagined objector reasons that if continuous (uninterrupted) development preserves an entity’s identity, then since *z* develops without interruption in both *Actual World* and in *W*_4_, *z* remains the same entity across the *Actual World* and *W_4_*. If so, then Lea = Mae, and, as a result, Lee’s argument for Developmental Plasticity fails.^17^

The Uninterrupted Development objection and zona-essentialism both attack Lee’s argument in the same way: by arguing that Lea = Mae. Since both approaches argue that Lea = Mae, therefore, we must ask whether Lee’s response to the Uninterrupted Development objection undermines zona-essentialism too. According to Lee, the Uninterrupted Development objection fails for two reasons. First, there is the possibility of chimerism, where two embryos fuse into one. The second, which I call the “Mad Scientist” problem, involves thought experiments in which embryos are artificially combined in strange ways. I consider each, arguing that even if Lee’s responses undermine the Uninterrupted Development objection, they pose no threat to zona-essentialism.

### The Problem of Chimerism

Regarding chimerism, Lee imagines two embryos, *f* and *m*, who are genetically very different. Imagine these embryos fuse, forming one embryo with two distinct genetic lines. Lee (2022) claims that if *f* supplies the embryo’s inner cell mass, then it develops into infant *F*. If *m* supplies the embryo’s inner cell mass, then it develops into infant *M*. Given that *f* and *m* have distinct genetic lines, it is possible that *f* contains two X chromosomes (making *F* biologically female) whereas *m* contains an X and Y chromosome (making *M* biologically male). Critically, the embryo’s development is never interrupted by any artificial means. So, the Uninterrupted Development objection would seemingly imply that *F* and *M* are numerically identical, “despite the fact that their bodies are composed of completely different cells and organs, and they are of opposite sexes” (Lee, 2022, 313). This, Lee claims, is implausible, and so, provides reason to reject the Uninterrupted Development objection.

One might insist that whenever fusion occurs, all “original” embryos cease to exist and that fusion gives rise to a new embryo altogether. For example, suppose that embryo *f* and embryo *m* combine to form embryo *fm*. If fusion destroys both *f* and *m*, then *f* ≠ *fm* and *m* ≠ *fm*. This remains true regardless of how *f* and *m*’s blastomeres feature into whatever developmental process *fm* takes. In other words, if identity does not survive fusion, then all infants from *fm* are distinct from infants that might have resulted from *f* and from *m*, respectively. Most importantly, this would mean that *fm* cannot develop into the same *F* as that which might develop out of *f* nor can *fm* develop into the same *M* as that which might develop out of *m*. The question would remain, however, whether any *F* that develops from *fm* is numerically identical to any *M* that develops from *fm*. Lee’s claim is that this is implausible.

Lee’s argument does not threaten zona-essentialism, even supposing it undermines the Uninterrupted Development objection. After all, zona-essentialism does not imply that *F* and *M* are numerically identical in *every* imagined case of chimerism. Reality is more complicated. Even in cases where zona-essentialism *does* imply that *F* and *M* are numerically identical, this result is not as strange as Lee supposes. Here is why.^18^

Suppose that when embryos *f* and *m* combine, they form embryo *fm*. With *fm*, there are two variables. First, the origin of *fm*’s zona. Second, the blastomeres that compose *fm*’s inner cell mass.

The first variable concerns *fm*’s zona and allows for three possibilities:

1. Blastomeres from *m* enter into *f*, while leaving *f*’s zona intact,
2. Blastomeres from *f* enter into *m*, while leaving *m*’s zona intact, or
3. Embryos *f* and *m* combine in such a way as to destroy the zona of both *f* and *m*.

The second variable concerns *fm*’s inner cell mass and allows for three possibilities:

- Only blastomeres from *f* form the inner cell mass of *fm*,
- Only blastomeres from *m* form the inner cell mass of *fm*, or
- Some combination of blastomeres from both *f* and *m* form the inner cell mass of *fm*.

These variables generate nine possible outcomes. If (1a) occurs, this gives rise to infant *_Zf_F*, meaning the zona from *f* is preserved and the resulting infant is genotypically and phenotypically *F-*like. If (1b) occurs, this gives rise to infant *_Zf_M*, meaning the zona from *f* is preserved and the resulting infant is genotypically and phenotypically *M*-like. And so on.

On zona-essentialism, plausibly, *_Zf_F* = *_Zf_M*. In this case, *f* is like an embryo that has undergone radical genomic editing. That is, embryo *f* remains the same organism throughout the process—the life of that individual organism persists—even though his or her genes are altered (even substantially) when blastomeres from *m* are integrated into his or her body. That said, zona-essentialism is also compatible with the view that *_Zf_F* ≠ *_Zf_M*. This is because it claims the zona is *necessary* for continued existence at the 16-cell stage, which is not to say that the zona is *sufficient*. In establishing sufficient conditions for the persistence of an embryo’s identity, one could argue that preservation of identity at the 16-cell stage requires that both the zona *and* a majority of an embryo’s blastomeres remain intact. So, defenders of zona-essentialism have options when assessing whether *F *=* M*, even if defenders of the Uninterrupted Development objection do not.

Still, assume that *_Zf_F* = *_Zf_M* (in at least some cases). Introduction of blastomeres from *m* provides *f* with far more ways of expressing itself (genotypically and phenotypically) than would have been the case otherwise. As noted above, this is akin to an extreme version of genetic modification that changes an embryo from only having, say, the possibility of developing dark hair to having the possibility of developing dark, blonde, red, or grey hair. When cells from *m* become integrated into *f*’s life (or vice versa), the ways in which the embryo can express itself expand dramatically. This process does not obviously disrupt the embryo’s identity. While it may seem odd to say that *_Zf_F* = *_Zf_M*—given how different they look as infants—this outcome is really just mundane genetics at work. An embryo’s genes typically include many possibilities for expression, such that infants and adults resulting from it will look quite different across different possible worlds. Chimerism is simply an extreme case, where, all else being equal, an individual’s possibilities for expression are multiplied in comparison to non-chimeric cases. This means that in chimeric cases, we should *expect* that the resulting differences in an embryo’s eventual expression will be much more varied across possible worlds than is typical.^19^ The increased range of ways in which the individual may express itself does not mean that resulting infants are numerically distinct from one another.

Now, suppose that (2b) occurs, which gives rise to infant *_Zm_M*. This means that the zona from *m* is preserved, and the resulting infant is genotypically and phenotypically *M*-like. On zona-essentialism, the following claims seem true: *_Zm_M* ≠ *_Zf_M* and *_Zm_M* ≠ *_Zf_F*. To unpack these claims, it is true that infants *_Zm_M* and *_Zf_M* are genetically and phenotypically very similar. One could stipulate that they might even be qualitatively identical. But on zona-essentialism, they are not numerically identical. With *_Zm_M*, blastomeres from *f* are integrated into the life of *m*. With *_Zf_M*, blastomeres from *m* are integrated into the life of *f*. In the first case, *m* becomes *fm* and *f* ceases to exist. In the second case, *f* becomes *fm* and *m* ceases to exist. Either way, given zona-essentialism, *m* and *f* remain numerically distinct embryos. So, *f* will give rise to an infant that is numerically distinct from any infant that develops from *m* (and vice versa). In the present case with *_Zm_M*, the life of *m* continues. With *_Zf_M*, however, the life of *f* would continue. Because *m* and *f* are numerically distinct, their resulting infants are also numerically distinct, *even though* both *_Zm_M* and *_Zf_M* are similarly *M*-like. Hence, on zona-essentialism, *_Zm_M* ≠ *_Zf_M* is true.

The takeaway is that zona-essentialism is not committed to the flatfooted idea that *F* and *M* are numerically identical in the same way that the Uninterrupted Development objection is. Rather, this discussion shows that the referents of *“F*” and “*M*” in Lee’s essay are ambiguous. “*F*” may refer to either *_Zf_F* or *_Zf_M*. Similarly, “*M*” may refer to either *_Zm_M* or *_Zm_F*. Once we disambiguate these claims, two things follow. First, there are grounds on zona-essentialism to argue that *F* ≠ *M* in many cases. It implies, for example, that *_Zf_F* ≠ *_Zm_M*. This implication aligns with Lee’s general intuition that *F* ≠ *M*. So, defenders of zona-essentialism are free to agree with Lee in some cases.

Second, in cases where zona-essentialism does imply something like the claim, “*F *=* M*,”—for example, when implying that *_Zf_F* = *_Zf_M*—this is less strange than Lee imagines. Assuming (for the sake of argument) that one embryo’s identity survives fusion, fusion dramatically expands that embryo’s capacity for expression. Unsurprisingly, when looking across possible worlds, the infants into whom this chimeric embryo can grow will vary from one another *far more* than is typical in non-chimeric embryos. That an individual being possesses an exponentially greater number of ways in which they may express themselves, however, does not imply that the individual’s numerical identity changes when their eventual expression takes one form or another. So, even if zona-essentialism is committed to the claim that *F *=* M* in some cases, this is not a concern.

To cover all bases, we should consider possibility 3 and possibility c (listed above). If 3 occurs, then both *f* and *m* cease to exist when fusion occurs. In these cases, fusion gives rise to a new embryo altogether, *fm*. In such a case, *f* ≠ *fm* and *m* ≠ *fm*. Still, given the available genes, *fm* may express itself in *F*-like ways, *M*-like ways, or as some combination of the two. No matter how *fm* eventually expresses itself, however, infants that originate from *fm* usually remain identical to *fm*.^20^ What is critical is that on zona-essentialism, when 3 occurs, *fm* is neither identical to *f*, nor to *m*. It follows that any infant resulting from *fm* is numerically distinct from those resulting from *f* and *m*, respectively. Hence, when 3 occurs, defenders of zona-essentialism are free to agree with Lee that *F* ≠ *M*, in that *_Zf_F* ≠ *_Zfm_M* and *_Zm_M* ≠ *_Zfm_F*.

As for possibility c, when blastomeres from both *f* and *m* form the inner cell mass of an embryo (whether that embryo be *f*, *m*, or *fm*), the resulting genotype and phenotype will more *F*-like, *M*-like, or some combination of them. The strangest cases involve (1c) and (2c). These cases illustrate why the zona is not sufficient for an embryo’s identity. Specifically, suppose that blastomeres from *f* form the entire inner cell mass of *m* (or vice versa). Next, for *reductio*, suppose that the zona is sufficient for an embryo’s identity to persist over time. Now, imagine that *_Zf_M* occurs and blastomeres from *m* manage to replace all of *f*’s blastomeres (so none of *f*’s blastomeres remain within *f*’s zona). If the zona is sufficient for identity, then *f* survives this process, despite losing all its blastomeres. In that case, *_Zf_F* = *_Zf_M*. This seems wrong. So, we should reject the claim that the zona is sufficient for persistence of an embryo’s identity. Alternatively, suppose that (1c) occurs, such that exactly one blastomere from *m* is integrated into *f*’s zona and inner cell mass, while all of *f*’s other blastomeres remain. It seems likely that *f* survives this process.

If the above suggestions are correct, then in cases where the zona remains intact, a 16-cell embryo’s loss of every blastomere alters its identity, but the loss of just one blastomere does not. The exact line—in terms of how many blastomeres are required for continuity of identity—is difficult, if not impossible, to draw. Miller and Pruss’s (2017, 538) “moderate genetic essentialism” is intuitive here. Moderate genetic essentialism holds that an organism’s identity can survive some moderate changes to its genetic makeup, but not any change whatsoever. To illustrate, imagine an embryo carries the BRCA1 gene, which predisposes her to developing breast and ovarian cancer later in life. Next, suppose we use gene editing to remove the BRCA1 gene and replace it with a benign gene. Seemingly, the embryo persists through this change. That said, more “substantial genetic changes,” Miller claims, “may not be identity-preserving” (2023, 228).

When it comes to gene editing and chimerism, we have a spectrum to consider, ranging from minor genetic changes which do not threaten identity, to major changes, which do. Again, drawing a clear, uncontroversial line along this spectrum—that separates survivable genetic changes from non-survivable changes—is probably impossible. Miller and Pruss themselves remain “skeptical that it is possible to give a comprehensive theory of what constitutes organismic identity” (2017, 539). Yet, that concession does not undermine the value of thought experiments as a means of providing clarity here. For present purposes, I need only assert that on zona-essentialism, a 16-cell embryo’s identity does not survive the loss of its zona. This does not mean that every change to a 16-cell embryo’s blastomeres is consistent with its survival. Some changes are survivable, others are not. Returning to Lee’s argument, we saw that on zona-essentialism, there are cases where *F *=* M*, cases where *F* ≠ *M*, and cases where it is difficult to tell whether *F *=* M*. So, even if the Uninterrupted Development objection problematically implies that *F *=* M* in all cases, this concern does not apply to zona-essentialism, which lends itself to a more fine-grained, careful analysis of chimerism.

### The Mad Scientist Problem

Lee’s (2022) second concern for the Uninterrupted Development objection is this. Consider a 2-cell embryo, *e*, which splits into identical twins, embryos *b* and *c*, respectively. Immediately after the split, *b* forms two blastomeres: *B_1_* and *B_2_*. Embryo *c* also forms two blastomeres: *B_3_* and *B_4_*. On reaching the 16-cell stage, all of *b*’s inner blastomeres derive from *B_1_* and all of *c*’s inner blastomeres derive from *B_3_*. Eventually, *b* develops into an infant, Betty, and *c* develops into another infant, Chloe.

In another possible world, *e* splits into *b* and *c*, but once *b* and *c* reach the 2-cell stage, a mad scientist intervenes. He plucks *B_1_* from *b* and plucks *B_3_* from *c*. Embryo *b* still contains *B_2_* while embryo *c* still contains *B_4_*. Both of those embryos continue developing uninterruptedly. The mad scientist then combines *B_1_* and *B_3_* into a new embryo, *d*. According to Lee, embryo *d* “may continuously develop without disruption into an infant *B*, whose cells, organs, and body are completely identical with those of Betty if [*B_1_*] yields the inner cell mass” (2022, 314). Alternatively, *d* “may continuously develop without disruption into an infant, *C*, whose cells, organs, and body are completely identical with those of Chloe if [*B_3_*] yields the inner cell mass.” Yet, Lee argues, “infants *B* and *C* are two distinct infants,” because we already saw that Betty and Chloe are numerically distinct (2022, 314). Thus, “*d* is numerically distinct from both *B* and *C*.” Since *d* can develop into either *B* or *C*, this means Developmental Plasticity holds for *d*. Thus, the continuous, uninterrupted development of *d* may give rise to one of two numerically distinct infants. If correct, the Uninterrupted Development objection fails.

On zona-essentialism, the Mad Scientist problem is a nonstarter. Beginning with embryo *e*, when twinning occurs, it is generally thought that *e* ceases to exist, giving rise to two new embryos, *b* and *c* (neither of which is identical to *e*).^21^ On zona-essentialism, if *e’*s zona is destroyed by fission, then any resulting entity cannot be identical to *e*.^22^ Next, we are told that *b* contains *B_1_* and *B_2_*, while *c* contains *B_3_* and *B_4_*. On zona-essentialism, supposing that *b* develops into an infant, it is plausible to argue that—whether *B_1_* or *B_2_* contributes to the inner cell mass of *b*—the resulting infant remains identical to *b*. That is, the whole organism, *b*, may express itself in different ways, depending on how its blastomeres are organized.

What happens when the mad scientist extracts *B_1_* from *b* and *B_3_* from *c* is a different matter. When that happens, suppose that *b*—whose inner cell mass now derives solely from *B_2_*—develops into an infant, Bethany. Likewise, *c*—whose inner cell mass now derives solely from *B_4_*—develops into an infant, Corey. On zona-essentialism, one can argue that Betty = Bethany and Chloe = Corey. This is because embryo *b* initially contains *B_1_* and *B_2_*, and so has the power to express itself as Betty—where the inner cell mass forms from *B_1_*—or as Bethany, where the inner cell mass is formed by *B_2_*. In Lee’s first world, *b* ends up expressing itself as Betty. In the mad scientist’s world, *b* expresses itself as Bethany. Despite these differences of expression, on zona-essentialism, Betty and Bethany plausibly remain identical across the two worlds. The same story holds for Chloe and Corey, respectively.

As for embryo *d*, on zona-essentialism, *d* is identical neither to *b* nor *c*. By extension, infants resulting from *d* are not identical to Betty, Bethany, Chloe, or Corey. Blastomeres *B_1_* and *B_3_* must be embedded into a “hollowed-out” zona (or, at least, a *de facto* zona) that comes from somewhere. On zona-essentialism, when this occurs, a new individual is created. In this regard, the creation of *d* resembles a teleporter case, where one entity is broken down into parts in a way that disrupts his or her identity, and then another entity is assembled from those parts (or, more accurately, assembled from some of the original’s parts combined with new ones). So, when *d* develops such that *B_1_* forms its inner cell mass, the resulting infant is *Betty-like* at most. We could even grant that the Betty-like infant (from *d*) is qualitatively identical to Betty (who grew from *b*). Given zona-essentialism, however, the resulting Betty-like infant is not numerically identical to Betty. Similarly, when *d* develops in such a way that *B_3_* forms its inner cell mass, the resulting infant is *Chloe-like* at most. Given zona-essentialism, the resulting Chloe-like infant is not numerically identical to Chloe.

Most importantly, once *d* is formed, *d* can express itself in a Betty-like or Chloe-like way. Since the Betty-like infant is not identical to Betty and the Chloe-like infant is not identical to Chloe, however, there is no problem when asserting that the Betty-like infant resulting from *d* is numerically identical to the Chloe-like infant resulting from *d*. Both these Betty-like and Chloe-like infants are different possible expressions of the same individual, *d*. This undermines two of Lee’s key claims. Contra Lee, it is false that Betty (resulting from *b*) is numerically identical to the Betty-like infant resulting from *d*. And it is false that Chloe (resulting from *c*) is numerically identical to the Chloe-like infant resulting from *d*. Hence, not only does Lee’s defense of Developmental Plasticity fail (again), but his response to the Uninterrupted Development objection poses no threat to zona-essentialism.

In sum, even though the Uninterrupted Development objection and zona-essentialism attack Lee’s core argument in similar ways, the latter avoids problems that may befall the former. A major lesson is that we must distinguish whole embryos from their parts. If, as I have assumed, the zona is an essential part of 16-cell embryos, then Lee’s argument for Developmental Plasticity, his defense of Developmental Plasticity against objections, and, subsequently, his main argument against ZAPO all fail. Obviously, the implications of his view (from Section II) would no longer follow either. Still, my response hinges on zona-essentialism, which I have taken for granted thus far. Zona-essentialism requires a defense, which is my focus now.

## VI. A DEFENSE OF ZONA-ESSENTIALISM

My defense of zona-essentialism begins with an explanation of why embryos are plausibly organisms. Doing so does not beg the question against Lee. This is because an alleged *implication* of Developmental Plasticity was that zygotes are not organisms. If, independently, we find that zygotes *are* organisms, then we have reason to reject Lee’s argument for Developmental Plasticity. In terms of simple first-order logic, showing that zygotes are organisms would counter Lee’s *modus ponens* with a *modus tollens*. Let us start, therefore, by discussing organisms (generally) before advancing an explicit defense of zona-essentialism.

### On Organisms

Organisms exhibit a specific kind of “integration” of “parts in the context of a coordinated whole” (Condic, 2008, 6). Whereas a mere collection of cells will “fail to exhibit coordinated interactions directed toward any higher level of organization,” an organism “acts in an interdependent and coordinated manner to ‘carry on the activities of life’” (Condic, 2008, 6). When examining a 16-cell embryo, we must ask: *at present*, does this entity exhibit “organization toward the production of a mature human body?” This is quite different from asking “which *parts* will serve as the material *basis* for a mature human body in the future,” as though the other parts are presently irrelevant to the organism’s identity.

Let us grant, for the sake of argument, that a 16-cell embryo’s inner cell mass serves as the material basis for its eventual infant-body. That does not imply that the inner cell mass is identical to the whole organism at present. Nor does it imply that the inner cell mass must remain unchanged to preserve the identity of the organism across time. Nor does it imply that the organism’s identity primarily (or exclusively) tracks the inner cell mass across time, space, and possible worlds. Instead, when it comes to identity, we should ask whether a given zygote, *z*, is the same *organism* (in toto) as the resulting infant, child, and adult.

With respect to the identity of living beings, the material basis for one’s body is likely not the whole story. If it were, then human beings would remain identical to their corpses after death. When an organism dies, however, it ceases to exist, despite leaving behind material remains. As Miller and Pruss write, there is a sense in which a “corpse is the same coarse-grained chunk of matter as the organism” from which it came, but corpses are not identical to organisms (2017, 537). So, at a minimum, to preserve identity across time, the matter that composes living beings must be integrated in the right way. This means that even if, across different possible worlds, different parts of a 16-cell embryo contribute to the material basis of its eventual infant-body, this does not imply that the *organism’s* identity changes across worlds. To say otherwise is to conflate an organism with some of its proper parts.

Lee’s mistake here is especially apparent when comparing embryos to blocks of wood. He writes,

… a 16-cell embryo is like a block of wood that is big enough to be made into two (or more) tables. When we have such a block, we can make either exactly one table using only, say, the left half of it or make two tables, one from the left half and another from the right one. And the production process of making a table from the left half is ontologically independent of the production process of making a table from the right one: The former process can yield the same table with or without the latter undergoing. So a numerically different table could have been made out of such a big block of wood depending on which portion of it is used. Likewise, when a zygote develops into a singleton, a numerically different singleton can develop from it depending on which six among the sixteen blastomeres are positioned inside and eventually yield the body of the resulting infant (Lee, 2022, 314-315).

True, a 16-cell embryo possesses enough *parts* to produce two or more infants. If we extract some parts of a 16-cell embryo, we can use them to construct more (numerically distinct) embryos. Given that blastomeres within a single embryo may rearrange on their own, a single embryo may *express itself* in a wide range of ways. As the above discussion of chimerism showed, however, there is a difference between expressing oneself in different ways and producing a change in one’s identity. The fact that an embryo may express itself in a variety of different ways does not imply that “a numerically different singleton” results when that occurs.

So, when thinking about 16-cell embryos as organisms (in toto)—where the right kind of integration presently holds between their inner cell mass, outer blastomeres, and zona—embryos are quite unlike blocks of wood, whose parts are not integrated at all. On this point, one anonymous reviewer observes that Lee’s central argument hinges on the intuition that infants, children, and adults are identical with just one proper part of 16-cell embryos. This may seem intuitive at a quick glance. For example, if I look at a photo of the 16-cell embryo that gave rise to me, I might see “myself” as equivalent only to the inner cell mass (while judging that the other parts merely keep “me,” the inner cell mass, alive). Similarly, when looking at an ultrasound photo of myself at 8 months gestation, I might identify “myself” in a way that ignores the umbilical cord and placenta to which “I” was merely attached.

When thinking about whole organisms, however, this is a mistake. As Kaczor helpfully points out, the fact that organs “are shed at a later stage of development does not entail that they are not parts of the human being at an earlier stage of development” (2015, 138). This means that “I,” the organism, may be composed of different parts (and different kinds of parts) at different stages in development. At the 16-cell stage, the whole organism is more than just its inner cell mass, even if the inner cell mass is especially important for future stages. Similarly, at 8 months gestation, the umbilical cord and placenta are parts of the entire fetal organism, even though those parts will be shed at birth. So, once we recognize that the whole organism *at a given time* is not identical with one of its proper parts—and that the parts of an organism may come and go in some cases without annihilating the organism itself—it becomes clear why Lee’s intuition is mistaken.

Of course, we can take parts from one embryo to make a new one. Recall, in *W*_2_, clinicians took parts from *z* to produce the embryo that became Shae, while leaving intact the original embryo (which later became Thea). When comparing the *Actual World* and *W*_1_, moreover, we saw that Lea—who developed from the undisturbed embryo—plausibly remained identical to Thea. If identity tracks an organism in toto—that is, the whole entity, whose parts are always integrated in the right kind of way at any given time—then Lea = Thea because both infants’ lives are continuous with the life of *z* in their respective worlds. During early development, total disintegration of the zona and all blastomeres never occurs in either case. So, on zona-essentialism, there is no reason to claim that either embryo’s identity is interrupted.

Further, Lea plausibly remains Thea even though the embryo that becomes Thea loses some of its parts. Given moderate genetic essentialism (discussed in Section V), an embryo’s loss of its outer blastomeres seems akin to an adult human being losing his own lungs and kidneys during a transplant. In either case, on receiving new parts, the identity of the recipient persists because their bodies integrate new (donor) parts in the right way. That is, in the imagined cases, the right sort of integration continues throughout each process. So, it makes sense to say that the life (and identity) of each organism continues, even when the processes involve the integration of foreign parts into their bodies. Finally, if identity tracks the life of the whole organism, rather than the continued existence of whatever material parts give rise to its body at future stages, then it is clear why Lee is mistaken when claiming that Mae = Shae. In fact, Mae = *Lea*, rather than Shae, because the right kind of organismic integration continues across time in both the *Actual World* and *W_4_* (which give rise to Lea and Mae, respectively). Shae, by contrast, develops after disintegrated embryonic parts are coupled with a new zona to produce a new (and so, numerically distinct) embryo.

I have claimed that what is critical to an organism’s identity across time is that its parts remain integrated in the right way. This does not say anything about the zona, however. For 16-cell embryos, a *de facto* zona is critical for this kind of integration and so, critical to these embryos’ identity as well. This brings us, at last, to an explicit defense of zona-essentialism.

### The Role of the Zona Pellucida

During the 16-cell stage, a de facto zona plays a crucial role in integrating an embryo’s (other) parts. Insofar as integration of parts contributes to something being an organism, therefore, the zona is critical to an embryo’s status as an organism. Moreover, if identity tracks the life of an organism (rather than the matter that gives rise to its body at later stages in life), then insofar as the zona is part of an embryo and partially sustains the existence of a 16-cell embryo (for a time), the zona is critical to its identity for a time as well. Here, then, are four reasons that support the claim that the zona is a critical embryonic part (the first of which includes an explanation of why we should regard the zona as a proper part of embryos in the first place).

### The Zona Is “Caught Up in the Life” of the Embryo

First, in terms of function, the zona is caught up in the activity of an embryo in important ways. As Condic details, within 30 minutes of the “sperm-egg fusion,” the zona undergoes significant “modifications” that “block [other] sperm from binding to the cell surface and prevent further intrusion of additional spermatozoa” (2008, 3). In this way, the behavior of the zygote differs from gametes. Whereas “the ‘goal’ of both sperm and egg is to find each other and to fuse, the first act of the zygote”—via modifications to the zona—“is immediately to prevent any further binding of sperm to the cell surface” (Condic, 2008, 3).^23^ These are substantial changes in function. When assessing whether an entity is an organism, we must see if its parts are integrated and coordinated in ways that promote the life and development of the whole entity. Not only does the function of the zona change completely at conception—from facilitating “sperm-egg fusion” to *preventing* it—but it does so in defense of the continued survival and flourishing of the whole entity.

One might respond that in this case, the *internal parts* of the embryo act upon the zona, changing its composition and function, while the zona itself does not actively produce these changes. This does not imply that the zona is not part of the entire organism, however. To illustrate, I may use my hand to comb through my own hair. In this case, an active part of my body is acting on an “inert” part (“inert” because hair is composed of dead cells). That one part of the body acts on another (inert) part, therefore, does not imply that the latter is not a part of my body. Perhaps the objector will insist that if something is “inert”—whether it be hair or the zona—then it *cannot* count as a body part. This strikes me as dubious.

From a practical, legal standpoint, courts have found that even though hair is “inert,” cutting someone’s hair without their consent is not only “common assault” but can amount to “assault occasioning actual bodily harm” (even absent all other kinds of physical contact).^24^ In practice, therefore, there is nothing obviously wrong about calling someone’s hair “part of her body.” Granted, what legally counts as a body part, may not count as a body part in biological or metaphysical terms. My point is simply that even if something is “inert,” that does not *obviously* preclude it from counting as a body part. In fact, any convincing account of bodily parthood must allow for the possibility that some body parts are inert (even dead). With gangrene, for example, one might need to amputate a dead limb to save a patient’s life. In such cases, it is sensible to say that a proper part of the organism has died and needs to be removed. If so, then even granting that the zona is “inert” and primarily acted *on*, this does not imply that it is not a part of the embryo.^25^

Some reasons to exclude the zona from being counted as an embryonic part have been addressed. This does not explain why the zona is part of the embryo, however, let alone an essential part. To that end, prior to conception, the zona is clearly part of an ovum. Following conception, the ovum ceases to exist and the zygote begins to exist. Subsequently, the remaining zona—which was a part of the ovum—is either a part of nothing or a part of something. If the zona were not a part of anything, it would seem best described as “the remains of a former ovum.” On this view, the zygote is like a hermit crab, while the zona is like the crab’s shell. This view understates the role played by the zona in embryonic life and development, however. In the minutes following conception, the zona, we saw, undergoes major physiological changes, designed to ensure the embryo’s survival and development. In this way, the zona is “caught up in the life” of the embryo, to use van Inwagen’s (1990) phrase.

Furthermore, when discussing the process of “generation,” van Inwagen writes, “*x is generated out of the ys at t* just in case that the *y*s come to compose *x* at *t* and *x* does not exist before *t* … and the *y*s do exist before *t*” (1990, 96). When this occurs, the *y*s that exist before *t* become part of *x* at *t*. Consider a standard case of conception, where a zygote begins to exist at some time, *t*. Prior to *t*, the zygote does not exist, but the atoms that compose some sperm and ovum do exist. van Inwagen notes that at the completion of conception, “the zygote is generated out of the atoms that had composed the sperm and the atoms that had composed the egg” even though “the zygote is not generated out of the sperm and the egg, since the sperm and the egg do not come to compose the zygote” (1990, 96). In other words, the sperm and ovum—as whole entities—are destroyed, and the atoms that composed them give rise to the newly formed zygote. Insofar as the zona—and so, the atoms that compose it—are caught up in the development and life of the embryo, therefore, it seems reasonable to say that they are part of the generative process.

Moreover, if integration and functional coordination are important marks of biological parts, then the zona meets these criteria. The modifications it undergoes are not coincidental but, instead, are ordered toward the survival of the whole entity. Not only does the altered zona prevent polyspermy (as noted above), but it also protects the embryo from “mechanical stress” as it travels through the fallopian tube. With that in mind, given the functional integration of the zona into the embryo’s life (and its role in the embryo’s survival), it seems reasonable to regard the zona as an essential embryonic part (for a time).^26^

### The Zona Protects the Embryo

Next, Siristatidis et al. observe that the zona “protects the embryos from mechanical stress prior to implantation” (2021, 2-3). That is, it plays an important role in holding the embryo together and protecting it from external threats as it develops and moves through its environment. Beckwith makes a similar observation, noting that “the zona pellucida … holds the embryonic cell-cluster together … for the benefit of the whole” (2007, 79). Thus, there are several ways in which the zona facilitates the survival of the whole entity in vivo. It would be odd to claim that the part of an entity that protects it from being ripped apart or destroyed (e.g., by the mother’s immune system) is somehow irrelevant to the integration of its parts. Supposing that the zona protects the general structure (and life) of the embryo, therefore, it clearly facilitates the integration of embryonic parts. If integration of parts is essential to something remaining an organism (not to mention, remaining alive), then it seems reasonable to regard the zona as an essential embryonic part.

Still, there is some possibility that “zona-free embryos” can survive under certain conditions. According to Yumoto, Shimura, and Mio, for example, “the [zona] is not always necessary for normal development” (2020, 1349). Perhaps worse, there appear to be cases in which the zona is an *impediment* to embryonic development and survival, where “artificial removal” of the zona (early on) might benefit some embryos.^27^ These cases deserve careful attention, lest their findings be overstated.

To start, Yumoto, Shimura, and Mio (2020) describe cases of normally-developing zona-free embryos, but elsewhere (and more recently), Yumuto et al. state that “in vivo, the [zona] is essential for avoiding immunological attack on zygotes and for preserving embryonic development during migration from the fallopian tube to the uterus. However, in vitro culture is not subject to immunological attack” (2024, 386).^28^ Indeed, as Nagatomo et al. observe, early-stage zona-free embryos (in mice) “do not develop when transferred to the oviduct” (2017, 1). Reconciling these claims is easy: all reported cases of zona-free embryos surviving have occurred *in vitro*, where the zona is not as “essential” to protect the embryo from various threats. This does not mean the zona is unimportant to embryos in vitro. As Nagatomo et al. observe, “the functions of the [zona] during *in vitro* culture include providing physical protection to the oocytes and embryos through to the blastocyst stage, serving as a physical barrier to viral infection, and promoting tight junction formation between blastomeres during compaction” (2017, 1). But, unlike zona-free embryos in vivo, zona-free embryos at least have a *chance* at survival in vitro.

When looking at hard cases for zona-essentialism, therefore, we are not considering any cases of embryos in vivo. The study by Yumoto, Shimura, and Mio (2020, 1349), moreover, focuses on “abnormally-fertilized oocytes (zygotes with three pronuclei),” which, Yalçınkaya et al. (2016, 95) report, are often “associated with spontaneous abortions after implantation.” This matters because, depending on the nature of a given genetic abnormality, there may be cases in which some products of conception are not actually human organisms at all.^29^ If there are such cases, therefore, they do not provide a clear counterexample to zona-essentialism. What I will suppose, therefore, is that there are cases in which conception occurs in vitro, a zona is not present during some of the earliest stages of development, and, nonetheless, an embryo, fetus, infant, and child develops.

Yumoto et al. (2024) report some such cases.^30^ In their study, some participants (the “zona-free” group) attempted to become pregnant using embryos whose zona had been removed artificially in vitro. The other participants (the “zona-intact” group) used embryos whose zona remained in place. Pregnancy rates for the zona-free group were 37.5% per patient and 24.3% per embryo transferred, while pregnancy rates for the zona-intact group were 5.9% per patient and 5.3% per embryo transferred.^31^ Thus, it seems that removal of the zona (early in development) is not only consistent with embryonic survival in some cases, but may even promote survival at times. Two responses are available to defenders of zona-essentialism here.

First, it might be that in these cases, *something* plays the role of the zona (to preserve identity), even if the zona itself is absent. For that reason, when describing zona-essentialism, I stated that a *de facto* zona is essential to the identity of early embryos. To illustrate, Nagatomo et al. explore the possibility of covering zona-free embryos “with a structure similar to that of the [zona],” namely “an agarose capsule” (2017, 1). The authors found not only that “embryos derived from these agarose capsules were able to develop normally,” but showed “good freezing tolerance” and an “extremely high” survival rate compared to zona-free embryos (Nagatomo et al., 2017, 1). Importantly, Nagatomo et al. worked with “oocytes without a zona,” so it is not the case that the zona was ever swapped out for any of these embryos (2017, 1).^32^

On zona-essentialism, one need only assert that the de facto zona—whether an actual zona, agarose capsule, or the surrounding medium in which embryos are manipulated—is essential to the identity of the organism (qua individual organism) during the earliest stages of development. If one is worried about the integration of foreign matter into an organism, as though the agarose capsule could not be *part* of the embryo, this is not unlike use of an artificial heart or kidney to sustain the integrating activity of an adult human being. So long as the artificial device is functionally integrated into the body of the organism in the right way, I see no reason to think it cannot become a proper part of the organism. What is more, Nagatomo et al. placed oocytes into their “capsules” *before* fertilizing them via intracytoplasmic sperm injection (2017, 2). Thus, the atoms that compose each capsule may be “caught up in the life” of the embryo in the same way that usually holds for the zona. The same may be said for the medium in which zona-free embryos are preserved in vitro.

The second response is a fallback position. For the sake of argument, suppose we have a case where there is no de facto zona whatsoever (i.e., nothing that performs the role of the zona and no medium surrounding the blastomeres that could reasonably be thought to do the work of the zona). Next, imagine that the blastomeres develop and are later implanted (and the process culminates with the birth of a healthy infant). On zona-essentialism, one can simply concede that there is a time at which an individual organism does not exist, as the zona-free cells that do exist form a Developmentally Plastic “whole” (meaning Lee’s arguments apply in these cases). To clarify, Mae et al. report that in zona-free cases that have been reported, embryos were created in vitro before having their zona “removed with a laser system” and blown away “like a jet car wash” (2023, i268).

Prior to the zona being removed, zona-essentialism likely implies that we have an individual embryo. Once the zona is removed, however—and if *nothing whatsoever* plays the zona’s role during the earliest stages of development (e.g., including the 16-cell stage)—then on zona-essentialism, it seems that the remaining embryonic parts are developmentally plastic. That is, they may develop into one of several numerically distinct individuals. The process described by Mae et al. (2023), therefore, begins with an individual organism, before that organism ceases to exist, even though its *parts* remain alive. And the parts that remain are a developmentally plastic collection of cells which may eventually form an individual organism.

Critically, even if one finds this concession to be counterintuitive, the concession poses no threat to ZAPO, which zona-essentialism is meant to defend. After all, ZAPO maintains that *most* born human beings are identical to the zygotes from which they originated. In these zona-free cases—where we are supposing, for the sake of argument, that there is no de facto zona whatsoever—resulting born human beings are not identical to the zygotes from which they originated. In this regard, such human beings are like identical twins, neither of whom is identical to the zygote from which they originated. That said, these zona-free cases are both highly contrived and occur less often than cases of twinning. Twinning poses no threat to ZAPO, however, so these cases pose even less of a threat.

### Zona-Essentialism Aligns with Parental Essentialism

Third, recent bioethical discussions consider the possibility of “three-parent embryos.” Three-parent embryos may be created when an intended mother carries a mitochondrial defect that may negatively affect the health of children derived from her oocytes.^33^ To avoid passing this defect onto her children, two embryos are created, one using a donor’s ovum and one using the intended mother’s ovum. The first embryo—created using the donor’s ovum and (perhaps) sperm from the intended father—contains healthy mitochondria. The second embryo—which is created using the intended mother’s ovum and the intended father’s sperm—does not. Thus, the process begins with creation of two human organisms. Next, nuclear DNA (from within the pronuclei of each embryo) is removed. As one reviewer observes, this “kills the embryos” but leaves most of their “components” intact. At this stage, nuclear DNA from the intended parents’ embryo is transplanted into remains of the donor embryo, where these “remains” include both the zona and other maternally-derived factors (such as mitochondria). This produces a new embryo, where its nuclear DNA comes from the intended parents and “essentialist maternal factors” come from the donor.

We might ask: is the embryo that exists after the transfer identical to either embryo that existed prior to the transfer? On the version of zona-essentialism I have defended—and according to those who say that the transfer “kills” both embryos—the answer is “no.” Given zona-essentialism and the assumption that at least some of an embryo’s blastomeres are necessary for his or her identity, this process involves the disintegration of two embryos followed by the assembly of a new embryo out of those disintegrated embryonic parts. “Three-parent embryo,” therefore, seems like an appropriate term on zona-essentialism, since we have a new embryo that originates from the gametes of three parents. Zona-essentialism actually explains why identity does not track the inner contents of an embryo alone (whether it be the embryo’s nuclear DNA or inner cell mass). This is an important result because it aligns with a commonly endorsed metaphysical thesis: parental essentialism.^34^

According to Miller and Pruss (2017), parental essentialism states that one’s identity is essentially linked to the gametes from which he or she originated. Three-parent embryos are the result of a different set of gametes than the respective gametes that gave rise to original two embryos. If, contra zona-essentialism, someone claims that the primary embryo retains its identity throughout the transfer process, then parental essentialism is false. After all, we would have to say that the resulting embryo originates from a different set of gametes (three or four, depending on the details) and that this change in origin does not alter the embryo’s identity. Since parental essentialism is intuitive, however, we should favor views that are consistent with it. Zona-essentialism is one such view, while views that identify embryos with only their inner content (whether nuclear DNA or inner cell mass) are not.^35^

### The Zona Provides a *Bona Fide* Boundary

Fourth, and finally, in recent debates over whether zygotes are organisms, one key feature of organisms is often emphasized: they must possess a specific kind of boundary or barrier that separates them from the outer world. As Smith and Brogaard put it, organisms are surrounded by a *bona fide* boundary, understood as “physical discontinuities in the usual sense of this term” (2003, 72). Bona fide boundaries differ from “fiat boundaries” which “correspond to no underlying physical discontinuities.” The edge of a table is a bona fide boundary, for example, while “postal districts” are fiat boundaries, in that they are arbitrarily imposed on objects or in space (Smith and Brogaard, 2003, 72).

The zona clearly provides a bona fide boundary. After all, it provides a bona fide boundary for the ovum of which it is a part. It would be odd to suggest that following completion of conception, that same bona fide boundary is no longer intact. If anything, following conception, the zona’s claim to being a bona fide boundary seems even stronger, given the protective role it plays and the changes it undergoes (e.g., becoming even more impermeable than before). Either way, if one necessary criterion for being an organism is possessing a bona fide boundary and the zona is what provides a clear, rigid boundary between the developing embryo and the outside world, then the zona seems essential to a zygote’s existence as an organism.

Against this, one may argue that the outer plasma membrane of the zygote and/or the collective border of its outer blastomeres are sufficient to constitute a bona fide boundary. If so, then whether the zona is present or not, the embryo would still meet the relevant criterion to count as an organism. Here, I offer two responses. First, it is not obvious to me that the borders of the blastomeres are sufficient to form a unified bona fide boundary around the entire organism. Rather, when looking at figure 1, it seems as though the blastomeres each possess their own bona fide boundaries, even though they are clinging to one another and interacting in intimate ways. The fact that the blastomeres may be rearranged or removed, specifically, suggests that there is not a collective, intact bona fide boundary surrounding the cluster of blastomeres. If so, then absent the zona, the blastomeres *by themselves* seem like distinct marbles that are grouped together.^36^ At least by comparison, the zona has a much stronger claim to being a bona fide boundary than the outer outline of the blastomeres.

Next, recall that when discussing three-parent embryos, the process involves creating an “enucleated” embryo, where the nuclear DNA of each embryo is removed, leaving behind the zona and other maternally-derived factors (including mitochondria, protein, and mRNA). One could argue that those maternally-derived factors—as well as the fluid that surrounds the blastomeres—are both critical parts of an embryo and that *they* somehow form a bona fide boundary around the embryo. On this view, one may assert that a bona fide boundary exists *within* the zona, which surrounds the blastomeres, but it is not the same as the collective outer boundary of the outer blastomeres. If such a boundary exists, it would seemingly remain whether the zona is present or not.

I admit that I do not see such a boundary. Were there one, however, then a defender of that view could respond to Lee’s argument in the same way that I do. Namely, they could argue that Lee has mistakenly identified whole embryos with some of their proper parts. I have given some reasons for thinking that the zona is an essential embryonic part. If one wishes to argue that some other part(s)—other than the blastomeres and zona—are essential (e.g., when providing the embryo with a bona fide boundary and preserving its status as an organism), then so be it. The outcome for Lee would likely be the same: his arguments fail. This, ultimately, is because Lee overstates the relevance of early embryos’ blastomeres to their identity.

## VII. OBJECTIONS TO ZONA-ESSENTIALISM

Zona-essentialism faces three major objections beyond those considered already. First, the zona is lost early in embryonic development. Not only do all embryos lose their zona, but they *must* lose it, if they are to survive.^37^ One may object that it is nonsensical to call part of an embryo “essential” when it is lost so early. Second, supposing that my arguments have shown that 16-cell embryos must possess *a* de facto zona during that stage of life, this does not imply that they must possess a *specific* zona. Only the latter view would seem to refute Lee’s arguments, however. Third, the zona comes from the ovum that contributes to an embryo’s formation at conception. Thus, zona-essentialism may imply that a given zygote is identical to the *ovum* from which it originates.^38^ This would be highly implausible. I address each objection.

### Zona-Essentialism and the “Hatching” Problem

First, zona-essentialism may seem false because the zona is lost early in embryonic development. As Sadler observes, the zona “disappears at the end of the fourth day” postconception (2019, 42). Specifically, the zona becomes permeable and eventually, the inner contents of an embryo squeeze through it in a process called “hatching.” If zona-essentialism is correct, then we might think that the zona cannot be lost without resulting in a change in the embryo’s identity. Thus, the objection goes, when hatching occurs, either the embryo dies and a new entity begins to exist *or* the embryo persists through this change, in which case, zona-essentialism seems false. Both implications would be problematic for any defense of ZAPO that relies on zona-essentialism.

In response, hatching does not undermine zona-essentialism. The zona is essential to the identity of an embryo *at the 16-cell stage* (and prior to that point), but not for the entirety of its life. Here is why. In terms of embryonic development, the zona is not lost until an embryo possesses around “50 to 150 cells” (Khan and Ackerman, 2023, 2). By this point, embryonic parts within the zona have become configured in such a way as to maintain a bona fide boundary—separating the embryo from the outer world—after the zona is lost. Thus, the embryo can maintain its status as an organism (and subsequently, its identity) in virtue of having this new boundary (the trophoblast). Hershenov’s (2023) presentation at Wake Forest University summarizes things well: the zona pellucida persists from fertilization to day four, at which point in time it disintegrates, and the trophoblast serves as a protective external barrier.

This process is not unlike what occurs when a crustacean molts from its shell or a snake sheds its skin.^39^ Before, during, and after the process of molting (or shedding), the organism maintains a bona fide boundary that separates it from the outer world, albeit using different parts at different times. A crustacean’s shell and a snake’s skin, moreover, are clearly *part* of the organism at some point in their existence. The shell (or skin) seems also to be a proper part of the organism even right before they are lost (i.e., when these parts are no longer required to preserve the organism’s bona fide boundary). In each case, organisms may begin with one boundary (whether an exoskeleton, skin, or the zona), before maturing within that boundary in ways that require shedding the outer boundary. So, even if an outer boundary is an essential part of an organism at one time—namely, prior to the entity generating a new boundary within its limits—that specific boundary may be replaced by certain processes.^40^ On zona-essentialism, the zona is essential to embryos during the first few days of development because it is seemingly part of what qualifies embryos as individual organisms in the first place.

In fact, the tables may be turned on our objector here, because given that the zona *must* be lost to allow for implantation (and so, survival of the whole entity), there is a real sense in which its dissolving at the right stage exemplifies the kind of coordinated activity between parts that is essential to organisms. Shedding of the zona, in other words, is not mere “happenstance,” as one reviewer puts it, but occurs as the result of integration between the embryos’ parts. Were the zona not integrated into the developmental process, in other words, its dissolution—in the right way, at the right time—would be mere coincidence. Such a view seems far less plausible than the claim that there are coordinated activities occurring between the zona and the other embryonic parts; activities that are ultimately directed at the survival of the whole organism.

Either way, the zona need not do the relevant work of integration for the entirety of an organism’s life. What is necessary to an organism’s identity across time is that its parts are continuously integrated in the right sort of way. How integration occurs (and via which parts) will vary with developmental stage. As Blackshaw and Rodger put it, there is a distinction between “determining if an entity is alive at *any* stage of development, and determining if an entity is alive at a *particular* stage of development” (2020, 550-551). What it means for a mature member of the species to exhibit organismic integration often looks very different from what it means for a member of the same species to display organismic integration at an earlier stage in life.

My claim is that a 16-cell embryo exhibits the right kind of integration partly in virtue of its zona, even if an adult human being exhibits the right kind of integration in virtue of some other parts (e.g., the brain or circulatory system).^41^ In typical cases of human development, integration remains a constant from the zygote stage onwards—and so, identity of the living being persists across time—even though the *means* of integration changes. Given that the parts responsible for organismic integration change over time, the parts essential to a human being *at a given time* are subject to change as well. The zona may be essential to zygotes and early embryos, but not essential to human organisms at every stage of development. So, even though the zona is lost early on in development, this does not undermine zona-essentialism.^42^

### Necessity of *some* Zona vs. Necessity of *the* Zona

Next, perhaps my claims in Section VI only show that *some* zona is essential to the identity of early embryos. This differs from showing that a *specific* zona is required. By comparison, imagine two possible worlds. In the first, my lungs perform their characteristic function naturally. In the second, a ventilator facilitates this function. Let us stipulate that were *nothing* to perform this function, I (the organism) would cease to exist.^43^ This means that my continued existence depends on something facilitating the function of my lungs, whether that thing be my lungs or some artificial means. From this, it would be a mistake to infer that in the second world, the ventilator is an essential part of my body (or even a proper part at all). In fact, the ventilator seems not to be a part of my body at all. By comparison, even if a de facto zona is essential to 16-cell embryos’ identity, one could argue that a *specific* zona is not essential in the same way.^44^

In response, a “zona substitution” seems quite unlike using a ventilator to facilitate respiration. The former is closer to a standard teleporter case, where a subject enters a teleporter, the matter that composes her is disintegrated, and matter—even the same matter—is formed into a materially identical “copy” of the original on the “other side” of the teleporter. Lee (2022) concedes that it seems highly unlikely that a subject’s identity persists through teleportation like this. The “copy” that exists at the end of the process may be qualitatively identical to the original, but not numerically identical to her. As for 16-cell embryos, when the zona is lost or replaced, this involves disintegration of the organism, even if only for a moment. Disintegration occurs in the teleporter case, but not with artificial ventilation. With ventilation, whether the brain or circulatory system is fundamental to organismic integration, relevant processes continue throughout the procedure. Hence, the organism’s identity persists throughout the procedure. We have, therefore, two types of cases in which an organism’s vital functions stop and start: one that results in the organism’s ceasing to exist, and one throughout which the organism continues to exist.

The trickiest cases for zona-essentialism occur when a 16-cell embryo’s blastomeres are removed from the zona and placed back into the same zona. Suppose this process occurs quickly. On zona-essentialism, as I have defended it, during the time that the blastomeres have been removed from the zona, the organism ceases to exist *qua organism*. When placed back within the zona, one of two things is possible: either the original organism is identical to the reintegrated organism or the resulting organism is not identical to the original. The former case would require allowing for the possibility of gappy existence of organisms, which, according to Toner “is the doctrine that an object can exist at one time, and then cease to exist, and then come to exist again with numerical identity” (2015, 255).

In these cases, the possibility of gappy existence does not seem troublesome. To illustrate, suppose we remove the brain of a healthy adult human for five seconds, then place it back within his body. It seems plausible that the organism ceases to exist during those five seconds and that the same organism exists after his brain is placed back into his body. After all, were we to take a snapshot of the world during the critical five seconds, we would not see the organism, but only his disintegrated parts.^45^ So, too, with an embryo, when his or her blastomeres are temporarily removed from the de facto zona in which they were previously contained. During that time, the embryo *as an organism* does not exist, even though his or her proper parts do. Alternatively, if one rejects the possibility of gappy existence, then in both the brain removal and blastomere removal cases, there is a new organism that begins to exist after the respective procedures are complete. Fortunately, zona-essentialism does not require a commitment one way or the other.

What about cases in which all 16 blastomeres are removed from their de facto zona and placed within a new one? Above, I argued that in these cases, the resulting organism is not numerically identical to the original. I argued this point, in part, because it is required if we maintain a commitment to parental essentialism (as described in Section VI). A different zona is derived from different parents (or, at least, different gametes) and so, the resulting organism—which is assembled of disintegrated embryonic parts—originates from different parents (or gametes), too. If so, then maintaining a specific de facto zona seems essential to a given embryo’s identity. To lose the zona that is (currently) integrating the entity’s parts is to disintegrate that entity, even if only for a short time. This process changes the entity from an organism to a collection of embryonic parts, even if only momentarily, which undermines the claim that its identity *as an organism* persists throughout the whole process.

### If I Was a Zygote, Then Was I an Ovum?

Lastly, as noted above, a zygote’s zona comes from the ovum that gives rise to the zygote. Thus, one could argue that that identity holds between a zygote and the ovum from which it originates. As Mills puts the objection, “if I was once a fertilized egg, then I was once an unfertilized egg” (2008, 327).^46^ After all, suppose that “the [ovum] blinks out” of existence “when the sperm breaches its cell wall” (Mills, 2008, 329). This is counterintuitive, Mills argues, since “cells don’t generally suffer annihilation when their outer layers are breached” (2008, 329). Instead, he claims, there is “no relevant difference” between “an amoeba’s absorbing its food and an [ovum’s] absorbing a fertilized sperm” (Mills, 2008, 329). Thus, “an [ovum] doesn’t cease to exist” at conception, “when a sperm breaches its cell wall” (Mills, 2008, 329). Rather, when seeing a sperm breach the cell wall, Mills argues, “the most natural description of these events” is that we have seen “one [ovum] *become fertilized*” rather than witnessing “the annihilation of one organism and the creation of another” (2008, 328). If so, then this seems like a *reductio* of ZAPO’s second claim (that most born human beings are identical to the zygotes from which they originated).

Drawing from Condic’s (2008) work, Stephen Napier (2017) points out several flaws in Mills’s argument. As Napier observes, during conception, modifications to the zona “prevent fertilization by more than one sperm” and so, “the new zygote loses a key property that the unfertilized oocyte had” (2017, 149). Importantly, Condic notes that when distinguishing between biological entities, biologists commonly look for changes in an entity’s “composition and behavior” (2008, 2). Not only are zygotes composed of different matter, but they behave radically differently from ova. As Napier puts it, zygotes take on “distinct potencies, behaviors, and composition” in contrast to gametes from which they originate (2017, 149). So, there are some reasons to think that zygotes are a distinct kind of thing from ova.

Still, suppose Mills is correct that “cells don’t generally suffer annihilation when their outer layers are breached” (2008, 329). This is compatible with the fact that *some* breaches of an organism’s outer layers do result in annihilation. A small pin prick, for example, can breach an adult human being’s outer layers without killing them. A cannonball fired through an adult’s chest, however, seems like the kind of breach that *will* end their life. The same holds for an ovum. Some breaches will annihilate the entity, others will not. What matters is how a given breach affects composition, behavior, and, above all, the integration and activity of the entity.

When a sperm breaches the ovum’s cell wall, we see a near-immediate change in cellular activity. Among other things, the primary end to which an ovum develops—being fertilized by a sperm—is now reversed.^47^ Thus, what we have is not a mere breach, as though the ovum were merely poked with a needle. Rather, we have a breach by a foreign entity, which quickly transforms the existent entity, forming a distinct, complex, and unified whole. Mills’s (2008) suggestion—that we simply watch the process under a microscope and describe what happens—is unhelpful here. The initiation of organismic integration is not something that the untrained eye will be able to spot easily. By comparison, Miller and Pruss argue, “looking at an egg being fertilized under an ordinary light microscope is similar to looking through a telescope at a person dying in her sleep far away—in both cases, the relevant change is not visible” (2017, 536). So, contra Mills, what *appears* to happen at conception is less relevant than what *does* happen.

Relatedly, the process initiated by conception is unlike an amoeba “absorbing its food.” Absorbing food allows an entity to continue functioning in the same way as before; integration of its parts is continuous throughout the feeding process. Conception, by contrast, gives rise to an entirely new set of functions (distinct from both sperm and ova).^48^ As such, following Napier at conception, the most “natural” interpretation is not that “one [ovum] *becomes fertilized*” (2017, 150). Instead, “the most natural description once we take into account the differences in powers, behaviors, and composition” is that conception gives rise to a new kind of entity altogether (Napier, 2017, 150). So, even on zona-essentialism—where the zona is necessary but not sufficient to an early embryo’s identity—we have sufficient reason to deny that zygotes are identical to the ova from which they originate.^49^ Despite sharing the same zona, ova and zygotes are substantially different things.^50^

## VIII. DEFENDING ZAPO

If zona-essentialism and ZAPO are correct, implications for the literature are significant. This is because zona-essentialism undermines many other philosophers’ arguments against ZAPO, including arguments by van Inwagen (1990), Smith and Brogaard (2003), and Kingma (2020). I consider each in turn.

### Problems for van Inwagen

**Fig. 3.**
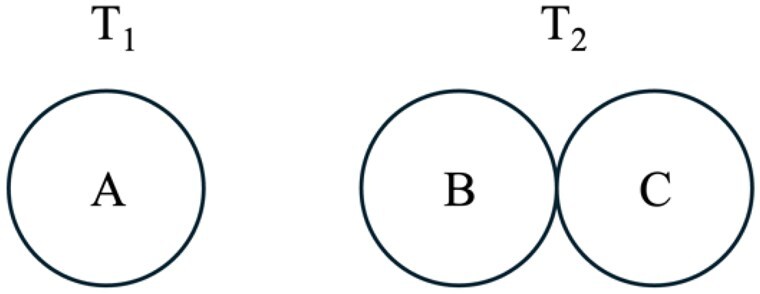
van Inwagen’s illustration of van Inwagen (1990, 152).

Like Lee (2022), van Inwagen (1990) confuses embryonic parts with whole embryos. First, he argues, “the zygote is a single cell” that “will divide mitotically, and the immediate result will be two duplicates of it that adhere to one another” (van Inwagen, 1990, 152). He illustrates this phenomenon with the following graphic, where T_1_ and T_2_ refer to distinct times (see fig. 3).

Entities “A,” “B,” and “C” are blastomeres and so, van Inwagen’s (1990) illustration suggests that embryos are composed entirely of blastomeres. If they were, then the transition from a 1-cell embryo to a 2-cell embryo would be exactly like twinning. As noted above, philosophers often claim that when twinning occurs, the original entity ceases to exist. So, when one blastomere divides into two, the original ceases to exist.^51^ If a zygote is identical to its single blastomere, therefore, then no infant (and no adult) is ever identical to a zygote. Instead, zygotes cease to exist the moment the first blastomere divides.

**Fig. 4.**
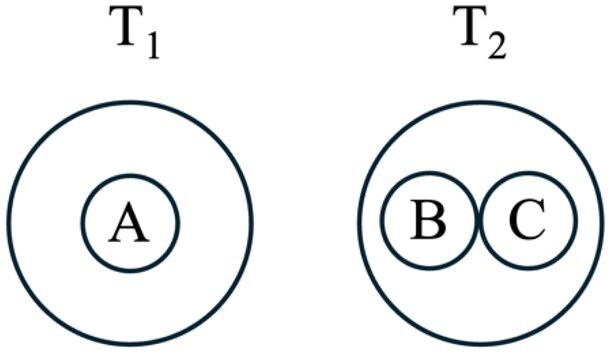
An Updated illustration of Mitosis.

In response, on zona-essentialism, embryos are not composed of blastomeres alone. Once we add the zona to the picture, mitosis looks closer to this (see fig. 4).

Given zona-essentialism, we can say that the zygote moves from having one major internal part at T_1_ to having two major internal parts at T_2_. As Napier puts this, “cell division by itself is not evidence that *the organism* has divided. Rather, the cells divide and eventually differentiate in order to maintain the survival and growth of the whole human organism” (2017, 151). In this regard, far from implying that zygotes are not organisms, the process of mitosis is a quintessential display of organismic integration. More carefully, even if the identity of the first internal *part* is lost when the blastomere divides, there is room on zona-essentialism to argue that the whole organism persists, nonetheless. So, contra van Inwagen, mitosis does not challenge ZAPO at all.^52^

### Problems for Smith and Brogaard

Smith and Brogaard (2003) make a similar error to van Inwagen. They claim that, as an embryo develops, “the inner cell mass will differentiate into two further tissues, only one of which will eventually become the embryo. The other will turn into extraembryonic membranes and tissue” (Smith and Brogaard, 2003, 61). In this passage, the word “embryo” incorrectly refers to an embryonic *part*, not the whole entity. To their credit, Smith and Brogaard anticipate this response, observing that “one may argue that the mentioned tissues are merely temporary parts of the embryo in much the same sense as baby teeth are temporary parts of the child” (2003, 61). They reject this view, however, on the basis that “it is not yet determined which parts of the inner cell mass are predestined to become embryonic cells” (Smith and Brogaard, 2003, 61).

This is an odd response. The view in question is that *all* the cells in play are, in fact, parts of the whole embryo. That some cells give rise to “extraembryonic membranes” is irrelevant. That is, even if it is not clear which blastomere (or which cells within the inner cell mass) provides the material basis for the embryo’s future infant-body, that does not make any of the blastomeres less a part of the embryo at present. Indeed, I have argued that at present, the embryo is composed of the zona and blastomeres within it. That the blastomeres within an early embryo may function in a wide range of ways speaks only to the many ways in which a single organism might express itself across different possible worlds. Still, Smith and Brogaard (2003) provide three objections.

The first objection stems from a discussion of fission. Smith and Brogaard claim that every zygote is “predestined to undergo fission” which “means that it will cease to exist almost immediately after it has been formed” (2003, 59). Thus, Smith and Brogaard identify a zygote’s single blastomere with the whole zygote. Following mitosis (as illustrated in fig. 4), they claim that “two zygotes [reside] inside the thin membrane” and so, they constitute “not one but rather two substances” (2003, 59). In so arguing, Smith and Brogaard make the same error as van Inwagen: mistaking a zygote’s single blastomere for the whole zygote. On zona-essentialism, we can grant that *part* of a zygote is “predestined to undergo fission,” but this does not mean that the whole zygote undergoes fission. In contrast, when whole embryos undergo fission, this just is “twinning.” Every zygote undergoes mitosis (as illustrated by fig. 4), but not every embryo undergoes twinning. The problem for Smith and Brogaard, at any rate, is mistaking the metaphysics of mitosis with the metaphysics of twinning. As noted in my response to van Inwagen, twinning involves one organism dividing into two new organisms, but mitosis does not.^53^

Second, Smith and Brogaard argue that, following conception, blastomeres are “kept together spatially by … the zona pellucida” and that “there is no causal interaction between the cells” (2003, 55). Kingma summarizes the concern nicely: “cells do not yet hang together in a way that forms a unified causal system” and so, cells “do not yet depend on each other in the right way” (2020, 373). If correct, then a zygote (and an early embryo) looks more like a “bag of marbles”—to use Kaczor’s illustration—rather than an organism (2015, 147). This seems like a profound understatement with respect to what the zona does, however. That there is “no causal interaction” seems mistaken, as the zona itself is significantly altered at conception and it *does* exert a causal force on blastomeres (e.g., by restricting their growth in ways that force them to change shape).^54^ Indeed, if nothing else, Newton’s Third Law of Motion applies, in that the pushing of blastomeres against the zona entails the zona pushing back on the blastomeres.

The idea that an embryo’s parts do not form “a unified casual system” is also suspect.^55^ As Beckwith notes, despite the fact that blastomeres are totipotent cells—and so, can develop into separate embryos under certain conditions—the fact that they do not do so, when contained within the zona, is compelling evidence that “the early embryo … behaves like a single organism with an intrinsic goal-directedness for which its cellular parts interact and communicate with one another” (2007, 79-80). Granted, integration observed in embryos is not nearly as complicated as in mature human beings, but as argued above, organismic integration often looks different—and involves different parts and levels of complexity—at different stages of an entity’s life. So, comparisons of early embryos to a disorganized “bag of marbles” are unpersuasive.^56^

Third, Smith and Brogaard raise the possibility of chimerism, arguing that sometimes, “one sperm fertilizes the egg and another fertilizes one of the other cells … separating at the time of the formation of the egg” (2003, 59). Later, “the two zygotes may then fuse to form a single individual.” For those who argue that “zygotes are already human individuals,” they must say that in these chimeric cases, “the two fertilized cell clusters are already two human individuals” even though “the final product of their fusion is like every other human being: it is one substance.” I have already explained why chimerism poses no threat to zona-essentialism (and ZAPO), however, so I will not rehearse those arguments here.^57^

### Problems for Kingma

Next, Kingma argues that “human organisms start, on average, roughly nine months after conception” (2020, 371). This is because, to count as an organism, an entity must have the right kind of “external boundary,” which separates it from the outside world. Following an embryo’s implantation into its mother’s uterus, however, there exists no such boundary between the embryo and its mother. As Kingma argues, “fetal and maternal-derived tissue cannot be easily separated; there is no line here, let alone the sort of boundary … marked by physical discontinuity in tissue” that organisms must have. Thus, until birth, embryos (and fetuses) appear to be parts of their mothers’ bodies (2020, 379).^58^

Kingma has little to say about *zygotes* and other preimplanted embryos. Suppose we grant that after implantation, there exists no “line” or “boundary” that separates embryos from their mothers’ bodies. It does not follow that prior to implantation no such boundary exists. Indeed, as argued above, the zona (and later, the trophoblast) *do* constitute a bona fide boundary. For this reason, Kingma concedes that “prior to implantation the arguments offered” here “become decidedly shaky” (2020, 384, n.5). Kingma’s “unconsidered and undefended” view, in this case, is that zygotes in petri dishes are not part of the petri dishes that house them, whereas “‘normally’ conceived embryos are part of the maternal organism,” even prior to implantation (2020, 384, n.5).

Granting that Kingma’s claim is “unconsidered,” it has some bizarre implications. Either a zygote in a petri dish is an organism, but it loses its status as an individual organism once it is placed in vivo *or* there are some other criteria (aside from possessing the right kind of boundary) that preclude *all* embryos from counting as organisms, whether they exist in vitro or in vivo. Kingma (2020) favors the former option, which regards zygotes in vivo as part of their mothers’ bodies even before implantation. This, Kingma claims, is because “the peritoneal cavity/uterus are most sensibly thought of as spaces internal to the maternal organism” (2020, 384, n.5).

This response is untenable. Kingma’s argument assumes “Smith and Brogaard’s ontological commitments,” which include the view that cavities within a mature human being’s body count as “external” to them (2020, 371). In this regard, Smith and Brogaard claim that human beings are akin to “tubes or hollow cylinders,” since our digestive tracts are, essentially, tunnels with an opening on either side (2003, 48). Similarly, there is an opening to the uterus through the vagina and cervix, which makes the uterine environment a cavity. On Kingma’s own assumptions, therefore, the environment in which a preimplanted in vivo zygote resides is topologically *external* to the mother’s body. Hence, preimplanted embryos in vivo are not part of their mothers’ bodies, even if implanted embryos become part of their mothers’ bodies.

One may hope for a more mundane response to Kingma (i.e., a response that does not require following Smith and Brogaard in thinking of mature human beings as “tubes”). Fortunately, there is one. Prior to implantation, the zona provides a bona fide boundary between the embryo and its mother. The trophoblast then takes over this role for a time. Once implantation occurs, moreover, Kingma (2020) is mistaken in claiming that no bona fide boundary exists between mother and embryo. As Hershenov (2026) explains, throughout development, there is a relevant boundary between an embryo and its mother at the fibrous connective tissue, first demarcated by the cytotrophoblastic shell, then a fibrous acellular connective tissue, Rohr’s layer and ultimately Nitenbach’s layer. Hershenov explains that this boundary takes on a variety of different features throughout development, but it still “acts functionally like a boundary (sealing and anchoring)” (2026, 227). In terms of sealing, Hershenov notes, the boundary ensures that “blood is never exchanged” between mother and child (2026, 228). Thus, Kingma’s claim—that there is no bona fide boundary between a mother and her implanted embryo—is false.

This means that Kingma’s main reason for claiming that embryos are not organisms is mistaken, as is her central argument that embryos and fetuses are part of their mothers’ bodies.^59^ Thus, ZAPO survives another attack. What is more, by appealing to the zona (and later, the trophoblast) as the embryonic parts that provide a bona fide boundary surrounding preimplanted embryos, we have a unified explanation of why embryos are distinct from both petri dishes and their mothers’ bodies. Kingma’s proposal, by contrast, treats embryos in vivo as metaphysically distinct from embryos in vitro (even though preimplanted embryos—in either environment—possess precisely the same kind of boundary and intrinsic properties). This is mysterious. If the zona provides early embryos with a bona fide boundary required for counting as organisms, such mysteries are avoided.

## IX. FROM THEORY TO PRACTICE

My defense of zona-essentialism—and ZAPO—is complete. I conclude by sketching some practical implications that follow (if my arguments are sound).

### ZAPO’s Practical Implications

First, if ZAPO is correct, then the bases for two kinds of anti-abortion argument are preserved. The first type of anti-abortion argument moves from claims about human beings to claims about the wrongness of abortion. Beginning at conception, human beings may be said to have some kind of inherent dignity, moral status, or other property such that killing them in utero is generally impermissible.^60^ This type of argument cannot succeed if the first conjunct of ZAPO—that zygotes are human organisms—is false.^61^ The second type of anti-abortion argument includes “future-like-ours” arguments.^62^ On these arguments, killing a human being is wrong because doing so deprives them of a future-like-ours. This line of argument requires that zygotes and embryos are numerically identical to the mature human beings they develop into. Hence, future-like-ours arguments depend on ZAPO’s second conjunct. Of course, defending ZAPO (and so, some of the bases of anti-abortion arguments) does not mean that either type of anti-abortion argument is sound. But if either type of anti-abortion argument is to succeed, then one (or both) of ZAPO’s conjuncts must be true. A defense of ZAPO, therefore, is a *partial* defense of these two types of anti-abortion arguments.

Second, if ZAPO is correct, then clinical practices and clinical language should reflect its veracity. At present, they do not. This is so, even though the scientific community does not seem to dispute the claim that embryos are organisms.^63^ To illustrate, consider current guidelines for the management of miscarriage or “unintended intrauterine death,” where embryos die in utero of natural causes. Current clinical guidelines routinely refer to embryos as “products of conception” (Prager, Micks, and Dalton, 2024; Law and Martin, 2020), a mere “group of cells” (UpToDate, 2024), “pregnancy tissue” (Tulandi, 2023), or just “pregnancies” (Griebel et al., 2005; Alves, Jenkins, and Rapp, 2023). If ZAPO is correct, then none of these euphemisms accurately describes embryos for what they are: human beings. Yet, the American College of Obstetricians and Gynecologists (ACOG) states: “we encourage people writing about reproductive health to use language that is medically appropriate, clinically accurate, and without bias” (2023, 1). If ZAPO is correct, therefore, there would be compelling reason to drop euphemisms when referring to zygotes and embryos.

Some medical experts may shirk at this suggestion, as evidenced by the fact that even now (and contra ACOG), they sometimes recommend *against* the use of perfectly accurate language in these contexts. Rodgers et al., for example, state that when it comes to first trimester pregnancies, words like “‘live’ and ‘living’ are best avoided because these terms may be appropriated by people outside of the field of medicine to support political rhetoric and proscriptive legislation” (2024, 5). In typical pregnancies, there is no disputing that embryos are *alive* during the first trimester. So, what we have is a group of medical experts citing political reasons in an argument *against* the use of scientifically accurate language. If referring to embryos as “living” is problematic, calling embryos “individual human beings” would likely be considered much worse, no matter how accurate this label might be. Medical experts cannot have things both ways, however. Either one uses accurate language or one relies on euphemisms to obscure reality. If ZAPO is correct, ACOG’s guidelines require endorsing the former option, no matter how politically inconvenient the truth happens to be. Alternatively, if ACOG changed its guidelines—and instead, recommended the use of politically expedient language, rather than accurate language—then current practices could continue, even were ZAPO proven correct.

Supposing that ZAPO is correct, there is another way in which its undermining of euphemisms would be a good thing. In the context of miscarriage management, euphemisms are often harmful to patients. Jessalyn Bohn, for example, writes “understanding the losses involved in intrauterine death through the lens of a ‘lost pregnancy’ or a ‘miscarriage’ misses the point for most bereaved parents: people mourning babies who die in utero are mourning the loss of their babies, not their pregnancies” (2021, 267-8). Similar claims saturate a recent special issue of *Narrative Inquiry in Bioethics*, which focused on “pregnancy loss.”^64^ ZAPO affirms the views of many bereaved parents: when intrauterine death occurs, an individual human dies. Recognition of this fact also explains why well-meaning advice to bereaved parents—such as “you can try again”—is unhelpful.^65^ “Trying again” does not raise a human being from the dead. If Bohn (2021) is correct, then acknowledging this fact may reduce patients’ disenfranchized grief.^66^ If ZAPO is proven correct, then acknowledging it as true would seemingly promote the well-being of many bereaved patients. Granted, the fact that explicit endorsement of ZAPO could reduce disenfranchized grief is not a sufficient reason to accept it as true, but this would be one fortunate implication.^67^

Third, if ZAPO were successfully defended, then practices that destroy zygotes and embryos would need to be reframed. This applies to clinical trials and to clinical applications of assisted reproductive technologies. Regarding clinical trials, consider the 14-day rule, which prohibits experiments on embryos that have developed beyond 14-days postconception.^68^ If ZAPO is correct, then one major argument for the 14-day rule is unsound. As Blackshaw and Rodger explain, the 14-day rule was suggested because the 14th day postconception marks “the point beyond which twinning was no longer thought to be possible” (2021, 712). Thus, it was inferred, “only after this could the embryo be regarded as a definite individual and a potential person—an entity with rights, such as the right to life” (Blackshaw and Rodger, 2021, 712). If ZAPO is correct, then this claim is false. A human being’s individuality and identity begins prior to 14 days postconception. Hence, one major justification for the 14-day rule would fail. Subsequently, we would need to consider whether the remaining reasons for the rule are sufficient to justify it.

In terms of research, if ZAPO is correct, then research that destroys zygotes and embryos kills human beings. There could be no denying this. The same goes for many assisted reproductive technologies where, in typical cases of IVF treatment, for example, individual human beings are discarded and destroyed for any number of reasons (including, but not limited to, possessing unfavorable genes, undesired sex chromosomes, etc.). That is, in a world where ZAPO is shown to be correct, there could be no denying that individual humans are routinely killed in the name of medical science and reproductive choice.

One may object that this suggestion—or, rather, the language I have used—makes relevant practices sound nefarious, when really, the practices are morally benign. The objector may reason that since “human” is usually synonymous with “human *person*,” my utterance sounds worse than it is.^69^ One could argue that it would be horrifying if human *persons* were treated in the same way that embryos are treated today. However, if embryos are merely human organisms (and not human persons), then, the objector may claim, killing them is not especially concerning.

In response, ZAPO is neutral on the question of embryonic personhood. As such, if ZAPO is correct, then the objection provides no actual reason to deny that human beings are routinely killed for scientific and personal gain. There is no need to resist this fact if one is convinced that “human being” and “human person” are distinct categories. That is, if routinely killing human beings involves no wrongdoing, then defenders of those practices have no reason to hide behind euphemisms in the first place. Instead, they should freely admit that the practices they endorse kill human beings, while explaining why this is permissible (e.g., because not all human beings are human *persons*).

Of course, if ZAPO is correct, then speaking accurately would mean the objector must realize that he is in the unenviable position of having to explain why his preferred account of “personhood” is not only correct, but that it should be enshrined within clinical and scientific guidelines. To elaborate, not everyone agrees that embryos are non-persons. Worse, as Christopher Kaczor (2015, 102) observes, when we look at history, “*every* previous division of humankind into two classes … in which one half was permitted to dispose of the other at will” is “nearly universally recognized as evil” (emphasis in original). So, if ZAPO is correct and the academic, scientific, and medical communities speak with accuracy—by openly acknowledging that widespread practices kill human beings (albeit not human persons)—then the burden falls upon them to explain why *this time*, the division of human beings into “person” and “non-person” categories is not morally monstrous.^70^

Perhaps a greater concern is this: if ZAPO is correct—and the purported permissibility of contemporary practices that involve destruction of embryos hinges on a specific account of moral personhood—then revealing this fact means having to admit that declarations by the medical and scientific communities (e.g., that certain practices are permissible or “good”) are rooted in something other than medicine and science. This is because the bases for such declarations are philosophical assumptions for which there is neither a medical nor scientific defense. Thus, whatever authority clinicians and researchers have (in virtue of their medical or scientific backgrounds) that authority counts for nothing when defending the morality of the practices in question. Confronting this truth would risk exposing something about people whose intellectual authority—and public credibility—derives from their medical or scientific credentials. They would be shown to have no actual authority (deriving from their own areas of expertise) to support assertions like “it is permissible to experiment on embryos” and “abortion is health care.”

This is not unlike Engelhardt’s challenge to those who work within bioethics. As Engelhardt (1996, 9) observes, “many provide bioethics consultations or advice as if there were one content-full bioethics, one canonical content-full bioethical orthodoxy, that should guide all secular moral decisions and justify all healthcare policy.” The problem for those who “claim to know *the* content-full secular moral vision” is that “there is no content-full bioethics outside of a particular moral perspective” (Engelhardt, 1996, 9). Thus, those who presume to speak for the “canonical” secular view—or even reason itself—are liable to be “imposing a particular moral vision, ideology, or moral orthodoxy” on others without any real authority to do so. In this regard, clinicians and researchers are no different from bioethicists. When they enshrine within public policy their own views of the organism/personhood distinction, they simply impose on others their own view (without any real authority to do so). Likewise, when medical experts endorse the destruction of human beings, they cannot be speaking for medical science because medical science is utterly silent on the matter.

Given these limitations, it is unsurprising when clinicians and researchers—like Rodgers et al. (2024)—resort to euphemisms or language games to obscure reality. If one’s own expertise cannot be relied on to justify the practices that one believes to be permissible (or even “good”), then one might wish to obscure this fact. Revealing it would mean having to justify oneself or, perhaps worse, might undermine one’s credibility in the public eye. Either way, if ZAPO is correct, then individual human beings are routinely killed in the name of research and reproductive autonomy. If clinicians and researchers continue to believe that killing human beings is permissible—whether doing so furthers our knowledge or allows patients to pursue their personal reproductive goals—then they should own these beliefs and speak accurately about them. Failure to do so, by contrast, only raises the suspicion that they have something to hide.

## X. CONCLUSION

In this essay, I have defended ZAPO: the claim that zygotes (and other embryos) are organisms and that generally, adults are identical to the zygotes from which they originate. In doing so, I have addressed objections from Lee (2022), van Inwagen (1990), Smith and Brogaard (2003), Mills (2008), and Kingma (2020). I argued that these authors often confuse whole embryos with proper parts of embryos. Correcting such errors renders ZAPO defensible. I ended by describing some practical implications that ZAPO may have, if proven true. If it turns out that zygotes are human organisms, then medical and scientific communities should not conceal this fact, whether via euphemisms or other language games. Indeed, I argued that at times, euphemisms are not only harmful to many patients (as is the case for many who experience unintended intrauterine death) but they also run contrary to calls from *within* the medical community for “accuracy” in language concerning reproductive care (ACOG, 2023, 1). Ultimately, if clinicians and researchers genuinely believe that killing human beings is permissible—and even *good*—euphemisms are unnecessary anyway. May medical experts speak with accuracy and work to justify their views openly.

## Footnotes

1 Smith and Brogaard’s view could be an exception, since they provide *sufficient* conditions for an entity counting as an organism and argue that embryos satisfy those conditions no later than 16 days postconception. This is compatible with thinking that embryos satisfy all necessary conditions for being an organism earlier than 16 days. That said, Smith and Brogaard (2003) reject this possibility.

2 “Most” because there are exceptions. Identical twins plausibly begin to exist after twinning has occurred, rather than at conception. See Pruss (2003) and Oderberg (2008).

3 See also Pruss (2003), Beckwith (2007), and George and Tollefsen (2008).

4 Lee himself makes a compelling case for why several mainstream critiques of ZAPO either fail or, at the very least, are highly “disputable” (2022, 317, n.21). Lee does so to elevate his own critique of ZAPO above others.

5 I intend “essential” in a metaphysical sense, such that, were an embryo to lose its de facto zona pellucida during its earliest stages, it would cease to exist as an individual organism. This is compatible with the fact that *parts* of an embryo can survive the loss of the zona pellucida during the earliest stages. In fact, surviving parts may even give rise to a distinct embryo under the right circumstances.

6 Following Lee, I use words like “we” and “us” to refer to individual members of the species, *Homo sapiens* (2022, 295, n.1). I do not use “we” to refer to persons in a Lockean sense, which would require possessing certain kinds of psychological properties or connections.

7 Also, for Lee, “human being” is synonymous with “human organism” or “member of the species *Homo sapiens*” (2022, 295, n.1). I treat these phrases as synonymous too.

8 Even if Marquis’s argument and those like it are vulnerable to Lee’s critique, some arguments—which conclude that killing zygotes is objectionable—avoid Lee’s critique. David Hershenov, for example, argues that killing zygotes is wrong because they “have interests in healthy development” (2023, 516). This, Hershenov (2023) argues, is true *even if* zygotes are not identical to the infants and adults that originate from them. If Hershenov is correct, then “pro-life” authors may reject the second disjunct of ZAPO, meaning Lee’s undermining of the second disjunct poses no problem for them. Still, if Lee (2022) is correct that the first disjunct of ZAPO is false—and so, it is false that zygotes are organisms—things become somewhat less clear for Hershenov’s (2023) view. If, to have interests, a living entity must be an organism, then zygotes do not have interests. On this assumption, Lee’s (2022) critique would threaten Hershenov’s (2023) view. Alternatively, if, to have interests, a living entity need only be alive (as zygotes are, whether they are organisms or not), then Hershenov’s argument seems impervious to Lee’s critique. This latter possibility comes with some costs, since it would require assigning interests to things like red blood cells. This will strike some readers as strange. But either way, I set aside Hershenov’s (2023) argument here, noting that it fares relatively well against some of Lee’s critiques (even if not all of them).

9 I suspect that Miller (2023) would favor this route.

10 One reviewer argues that when Lee talks about making “eight copies of *B_8_*,” this “reflects an unrealistic view of ‘identical’ blastomeres” since the copies are “ontologically and biochemically distinct” from *B_8_*. In Lee’s defense, his argument does not rely on the claim that the copies of *B_8_* are numerically identical to *B_8_*. At most, they might be *qualitatively* identical to *B_8_*. But either way, this does not affect Lee’s central argument, which relies on the claim that identity of the individual tracks the inner cell mass. Lee’s point is that the individual’s identity is seemingly unaffected in cases where the inner cell mass remains unperturbed, whether the inner cell mass is coupled with the “original” outer blastomeres or with manufactured copies of the original outer blastomeres. So, we need not attribute to Lee the claim that copies of *B_8_* are identical to *B_8_*.

11 Lee considers several other possible worlds to defend various tangential points. In *W_3_*, for example, the events of *W_2_* are almost the same. The difference is that after *B*_9—_*B*_16_ are removed, blastomeres *B*_1—_*B*_7_ are destroyed. Hence, in *W_3_*, the infant “Thea” is never born. Preventing Thea from coming into existence does not affect Shae’s existence, however, which shows that Thea and Shae are ontologically independent. See Lee (2022).

12 These points by Lee make clear why developmental plasticity does not apply to adult human beings, even supposing that a given adult may develop differently in the future (across different possible worlds). For adults, it is false that their possible future selves grow entirely out of different “matter, cells, and organs.” So, even if there are different possible futures for how adults might develop, it does not follow that adults are developmentally plastic.

13 See Hershenov (2026) and Sadler (2019).

14 To be clear, when talking about “parts” of an embryo, I refer to its “proper parts,” where *X* is a proper part of *Y* just in case *X* is a part of *Y* and it is false that *X* = *Y*. See Varzi (2019).

15 There are reportedly cases in which zona-free embryos have grown into healthy fetuses and newborns, despite lacking a zona. See Yumoto et al. (2024). I address these cases at length the subsection titled, “The Zona Protects the Embryo.”

16 For an overview, see Saey (2016). I also discuss three-parent embryos in detail in the subsection titled, “Zona-Essentialism Aligns with Parental Essentialism.”

17 Relatedly, development is disrupted in *W_1_* and *W_2_*. Hence, Lee’s objector may argue that entities in the *Actual World* and *W_4_* (namely Lea and Mae) are numerically distinct from entities in *W_1_* and *W_2_* (ie, Thea and Shae). If so, then Lea = Mae and Lea ≠ Thea. The central dispute, however, is whether Lea = Mae.

18 To be abundantly clear, in what follows, I am considering cases in which fusion (and resulting chimerism) occurs prior to the zona being lost. This is because Lee (2022) mentions this possibility explicitly. One reviewer observes, however, that in nature, fusion and chimerism only occur *after* the zona has been shed. Loss of the zona, the reviewer notes, results in distinct embryos being able to “interact with each other.” Fortunately, what I say in my defense of zona-essentialism is informative with respect to these cases, too. In short, either fusion (after the zona is gone) results in the loss of identity or it does not. If fusion destroys identity, then it follows that any resulting infant from the fused embryo is numerically distinct from infants that might have developed from the original two embryos. If there are cases in which the identity of one embryo survives fusion, however, then consistent with what I argue below: the cells from one embryo may be integrated into the life of the other (or vice versa). This expands the ways in which the fused embryo may express itself (well beyond the norm), but this is not as strange as Lee suggests.

19 One reviewer notes that some chimeric adults may have one set of DNA in a localized part of their body (e.g., their tongue), while having another set of DNA in the rest of their body. If so, then the “amount” of genetic information that comes from one embryo or the other will vary along a spectrum. As I argue below—when discussing a view called “moderate genetic essentialism”—sometimes the introduction of foreign genetic information into an embryo is consistent with the embryo’s retaining its identity and sometimes it is not.

20 “Usually” because we could imagine *fm* twinning, in which case, infants that “arise from” *fm* would not be identical to *fm*, but would be identical to the two (or more) embryos into which *fm* divided, respectively.

21 See, for example, Pruss (2003) and Oderberg (2008).

22 One alternative would be to argue that the zona persists through this change, in which case, the original entity may be identical to whichever resulting embryo maintains the original zona. Fission, in this case, would be a form of “budding,” where one twin generates another. See Playford (2020). Since it does not affect my argument against Lee, I do not consider the possibility of budding here.

23 See also Sadler (2019).

24 See Judge and Cresswell (2006).

25 A reviewer worries that if dead cells, like hair, count as a body part, then “getting a haircut would be a form of self-mutilation.” This assumes that all bodily modifications are a kind of mutilation, which is false. If I whiten my teeth, pierce my ears, or undergo an appendectomy, my body is modified (even cut open and cut apart) but no “mutilation” occurs. So, cutting one’s hair involves a kind of bodily modification, sure, but it is not a form of mutilation.

26 As one last objection, one might think I have proven too much. One reviewer argues that on implanting into the mother’s body, the embryo “alters the structure and function of the maternal endometrium,” but this does not imply that the endometrium is part of the embryo. I tend to agree, though Yancey (2020) does raise the question of whether, at implantation, the mother’s body becomes “part” of the embryo. In response, however, the zona (and the atoms composing it) were part of something that no longer exists following conception (i.e., the ovum). The thing of which they were a part is gone, and so, when they become caught up in the life of the embryo, it seems they become part of the embryo (if anything). The endometrium, as part of the mother, is different, in that the thing of which it is a part (the mother) exists before and during implantation. So, while we might say that the implanted embryo uses its mother’s body, this does not mean that the atoms that compose its mother’s bodies are caught up in its life (in the same way). For a detailed look at the metaphysics of implantation and pregnancy, see Hershenov (2026).

27 See Mae et al. (2023) and Yumoto et al. (2024). I am grateful to an anonymous reviewer for raising this point.

28 Lest the reader think that I am using brackets to manipulate this quote, “zona” is in brackets because the authors I am quoting tend to use “ZP” for “zona pellucida.”

29 For an overview of this discussion, see Colgrove (2023).

30 This study was on couples who struggled to conceive, given that their embryos were prone “to excessive fragmentation.” So, we should not take these cases as representative of the norm.

31 See also Mae et al. (2023).

32 Notably, zona-free oocytes are typically “discarded,” the authors explain, because “it is difficult to prevent polyspermy at the time of fertilization and they are difficult to handle without damaging them” (Nagatomo et al. 2017, 1) This reinforces the importance of the zona, even in vitro.

33 I am indebted to an anonymous reviewer for the description of three-parent embryos as it appears in this paragraph and the next.

34 See Kripke (2001).

35 One could respond that if the embryo is identical to the nuclear DNA that is transplanted, then in cases of “three-parent embryos,” the resulting embryo originates from the same parents as it did prior to the transfer. That is, one could argue that the nuclear DNA that is transplanted is “the whole embryo” and that it comes, essentially, from a specific sperm and ovum. On this view, when the transfer occurs, one would have to say that “a whole embryo” is removed from one zona and placed into another. There are some costs to this view. For one thing, “three-parent embryo” would be a misnomer, since we would have a *two-parent* embryo that is removed from one zona and placed into another. Worse, it would require denying that the donor gametes play a causal role in the origin of the resulting infant, which seems false. The view would also require denying the claim that the process “kills” both embryos (since one of the embryos is said to survive). Thus, we would need an explanation of how nuclear DNA can remain adequately integrated to count as an organism—not to mention the *same* organism—throughout the transfer process.

36
Kingma (2020) makes this same kind of argument, though it has received a fair share of scrutiny. I return to this analogy in Section VII, where I reject Kingma’s view.

37 As Condic (2022) observes, shedding the zona is part of what allows embryos to implant in utero.

38 Both Mills (2008) and Chaffer (2023) advance this type of objection.

39 For an explanation of the molting process, see Alaska Fisheries Science Center (2022).

40 “Essential” in this context is understood in the sense that, when an organism’s outer boundary is lost entirely—leaving nothing to “hold its parts together”—the organism ceases to exist (even if its parts survive).

41 Which part(s) of the adult body are critical to organismic integration is debated. See Brown (2019) for a discussion.

42 As one anonymous reviewer observes, there is nothing odd about saying that time-indexed properties, such as “having a zona” apply to human beings at a given moment in their lifespan. For example, during the earliest stages of development, human embryos have tails. In fact, Sadler (2019) suggests that *lacking* a tail during the first 50-56 days of development would be abnormal. The time-indexed property of having a de facto zona is no more exotic than time-indexed properties of having a tail, having a placenta, or having baby teeth at a given time. One might wonder about the distinction between time-indexed essential properties and time-indexed accidental properties, of course. On the view I have defended, it is essential to an organism that its parts be integrated in the right way at any given time and the means of integration may vary over time. At certain stages of life, the means of ensuring organismic integration may be limited. For example, suppose only a de facto circulatory system can accomplish this in adult human beings. If so, then having a de facto circulatory system is a time-indexed essential property for adult humans. That is, at the later stages in life, humans must have the property of having a de facto circulatory system, or they will cease to exist. If a de facto zona is required for organismic integration at the 16-cell stage, therefore, then it seems like a time-indexed essential property. By comparison, having baby teeth is a time-indexed accidental property of many human beings.

43 This allows us to bracket worlds in which I am, say, cryogenically frozen (and so, plausibly alive in some real sense despite the fact that respiration is not occurring).

44 I am grateful to an anonymous reviewer for raising this objection.

45 Importantly, this is compatible with thinking that the human *person* in a Lockean sense exists throughout the entire process, since the brain and any relevant psychological states (or properties) may remain unperturbed.

46 Note that “fertilized egg” a misnomer. See Condic (2020) and Miller and Pruss (2017) for an explanation.

47 cf. Condic (2008) and Napier (2017).

48 This also suggests why Mills’s comparison of zygotes to bacteria is unsuccessful. In both cases, Mills (2008, 333) claims, an entity (whether bacterium or ovum) may survive alteration of its genetic code. This may be true for ova, in that it is plausible to believe that gene editing does not alter the identity of a given ovum. However, at conception we do not have the *mere* alteration of an entity’s genetic material as though all else is equal.

49 To be clear, if one were to argue that the zona is sufficient for an entity’s identity, then Mills’ (2008) criticism would likely succeed. This provides even more reason to reject the claim that the zona is sufficient for identity.

50 For another version of Mill’s objection and a response, see Chaffer (2023) and Blackshaw (2023), respectively.

51 cf. Mills, (2008).

52
Mills (2008) makes the same argument as van Inwagen. My response here applies just as well.

53 As for the possibility of twinning itself, Smith and Brogaard (2003) are ambivalent on whether the potential to twin implies that an entity is not an organism. They claim that we can accept that the potential for twinning does not discount the embryo from counting as an organism, but “only at the price of allowing that human beings, like amoebae [and] flatworms … can contain within themselves the potentiality for division” (Smith and Brogaard, 2003, 69). The alternative, Smith and Brogaard state, is to “insist … that the type of unity” required for an entity to count as an individual organism “excludes this potentiality” (2003, 69) Importantly, whichever option one selects is irrelevant to their overarching goal of showing that human beings begin to exist “*no later* than sixteen days after fertilization.” This means that Smith and Brogaard have no real reason to claim that twinning precludes an entity from counting as an individual organism; doing so is tangential to their thesis. This is fortunate, since as Pruss argues, it “is plainly false” to assert that “if it is merely possible that an organism will split in the future, then we do not have a genuine individual on the scene” (2003, 176). That amoeba and flatworms are individual entities with “the potentiality for division,” in other words, is an exceptionally low “price” to pay. cf. Oderberg (2008) on these points.

54 In a direct response to Smith and Brogaard (2003), Damschen, Gómez-Lobo, and Schönecker also highlight the role of the zona—albeit, emphasizing its biological importance, rather than its metaphysical importance—writing, “S&B arguments *entirely* rest upon the assumption that the zona pellucida plays no substantial role and that there is no internal causal interaction in the early embryo,” yet “the zona pellucida *does* indeed play a substantial role and that there *is* internal causal interaction in the early embryo” (2006, 175, n.13).

55 In this essay, my focus has been more on metaphysics than on biology, but for more details on why early embryos are a unified causal system in the biological sense, see Damschen, Gómez-Lobo, and Schönecker (2006) and Koch-Hershenov (2006).

56 See also Kaczor (2015) who explains why this comparison is misleading.

57 See Section V, under the heading “The Problem of Chimerism.”

58 One reviewer observes that Kingma’s discussion, in this instance, seems illogical. This is because Kingma’s claim that “fetal and maternal-derived tissue cannot be easily separated” presumes that there is a distinction between fetal and maternal-derived tissue in the first place (2020, 379). If so, then it seems like Kingma is saying we cannot separate two things that we already know to be distinct. As argued in the next footnote, that two things are difficult to separate—or even *practically impossible* to separate—does not imply that there is no bona fide boundary between them, nor does it imply that they are one and the same thing.

59 Against this, Kingma argues that even if there *is* a boundary where Hershenov suggests, “it is traversed by ‘tubes and canals’ through which blood and nutrients flow” (2020, 380). Yet, as Hershenov emphasizes, “blood is never exchanged,” so the embryo’s circulatory system is not an extension of the mother’s (2026, 228). Instead, Hershenov explains, “one can liken it to a tangle of charger cords: difficult to undo, but the cords are discernable and separable” (2026, 228). Contra Kingma, therefore, even though it would be difficult to separate an implanted embryo from its mother (while leaving all boundaries intact), it is false that there is no bona fide boundary between the two.

60 See, for example, arguments by Beckwith (2007), Kaczor (2015), and Miller (2023).

61 Once again, when following Lee I assume that “human organism” is synonymous with terms like “human being,” and “individual members of the species, *Homo sapiens*” (2022, 295, n.1).

62 See Marquis (1989) and Colgrove (2019).

63 See Condic (2013) and, for a sample of excerpts from textbooks, see Snyder (2017).

64 See DuBois, Iltis, and Walsh (2022).

65 cf. Lancet, where the author observes that the “just try again” mindset that is “enshrined in many national guidelines … underestimates, and risks dismissing, the personal physical and mental consequences of a miscarriage” (2021, 1597).

66 What about patients who do *not* regard embryos as human beings? Would it not be harmful to say to these patients that “a human being has died”? Again, if we follow ACOG’s (2023) advice, then medical and scientific communities should strive for *accuracy* in their language. If ZAPO is proven correct and some patients insist that embryos are not organisms, they are simply wrong. How best to handle patients who deny ZAPO, in that case, would be like any other case in which patients’ beliefs are mistaken. Either way, one may approach the matter delicately, but without endorsing the patients’ misunderstandings of reality.

67 Similarly, any tendency of anti-ZAPO assertions—when it comes to worsening disenfranchised grief—is an unfortunate implication of those views, but not a sufficient reason to reject them.

68 See Warnock (1984).

69 For a classic overview of the distinction between human organisms and “persons,” see Mary Anne Warren (1973).

70 Not only that, but there are philosophical puzzles for those who argue that there is a meaningful distinction between human organisms and human persons. Suppose, for example, that someone thinks a human organism is a material, biological entity, while a human person is the immaterial soul (or mind or psychological states) that somehow exists within the body of a human organism. In that case, Pruss observes, assault seems akin to “a mere property crime” (2003, 173). The immaterial soul is never touched—nor are psychological states touched—even if the body is damaged or destroyed. If the person is identical with their soul (or collection of psychological states, etc.), therefore, the human person is never touched. This is deeply counterintuitive. As Pruss rightly argues, there is probably “too much absurdity” here (2003, 173). Either way, insofar as they claim that killing human beings in the name of scientific progress (or reproductive autonomy) is permissible, members of the medical and scientific communities must not only contend with Kaczor’s (2015) moral challenge but must also defend their preferred account of the organism/person distinction against metaphysical challenges. As none of this is the work of science or medicine, experts within those fields cannot reasonably invoke their own credentials to settle these disputes. Instead, they must depend on rigorous philosophical arguments to have *any* chance of justifying their views.
